# A postsynthetically 2’-“clickable” uridine with arabino configuration and its application for fluorescent labeling and imaging of DNA

**DOI:** 10.3762/bjoc.13.16

**Published:** 2017-01-20

**Authors:** Heidi-Kristin Walter, Bettina Olshausen, Ute Schepers, Hans-Achim Wagenknecht

**Affiliations:** 1 Institute of Organic Chemistry, Karlsruhe Institute of Technology (KIT), Fritz-Haber-Weg 6, 76131 Karlsruhe, Germany; 2 Institute of Toxicology and Genetics, Karlsruhe Institute of Technology (KIT), H.-v.-Helmholtz-Platz 1, 76344 Eggenstein-Leopoldshafen, Germany

**Keywords:** dyes, fluorescence, nucleic acid, oligonucleotide

## Abstract

The arabino-configured analog of uridine with a propargyl group at the 2’-position was synthesized and incorporated into DNA by solid-phase chemistry. The fluorescence quantum yields of DNA strands that were postsynthetically modified by blue and green emitting cyanine-styryl dyes were improved due to the arabino-configured anchor. These oligonucleotides were used as energy transfer donors in hybrids with oligonucleotides modified with acceptor dyes that emit in the yellow-red range. These combinations give energy transfer pairs with blue–yellow, blue–red and green–red emission color changes. All combinations of arabino- and ribo-configured donor strands with arabino- and ribo-configured acceptor strands were evaluated. This array of doubly modified hybrids was screened by their emission color contrast and fluorescence quantum yield. Especially mixed combinations, that means donor dyes with arabino-configured anchor with acceptor dyes with ribo-configured anchor, and vice versa, showed significantly improved fluorescence properties. Those were successfully applied for fluorescent imaging of DNA after transport into living cells.

## Introduction

The “click”-type reactions [1], in particular the 1,3-dipolar cycloaddition between alkynes and azides (CuAAC) is a broadly applied strategy for postsynthetic oligonucleotide modification since both reactive groups are not present in nucleic acids [2–5]. Although Huisgen described the uncatalyzed reaction yielding 1,2,3-triazoles already in the 1960s [6], the bioorthogonality with respect to proteins and nucleic acids emerged after Sharpless [7] and Meldal [8] had reported that catalysis by Cu(I) enhances not only reaction rates but improves also regioselectivity. The formation of oligonucleotide oxidation side products by Cu(I) is avoided by the use of chelating Cu(I) ligands, in particular tris[(1-benzyl-1*H*-1,2,3-triazol-4-yl)methyl]amine (TBTA) and better water-soluble derivatives [9–10]. The CuAAC cannot only be applied for conventional postsynthetic oligonucleotide modification in solution but also on solid phase [11] and for the introduction of multiple postsynthetic modifications [12]. The azide groups for CuAAC are typically placed onto the fluorescent dyes since azides are not compatible with phosphoramidite chemistry. The alkyne groups as reactive precursors are attached to the oligonucleotide [13], especially at the 5-position of pyrimidines [13], the 7-position of 7-deazapurines [14], and the 2’-position of ribofuranosides [11,15]. These positions were chosen since they are typically accepted by DNA polymerases in primer extension experiments and PCR [4,16].

**Scheme 1:**
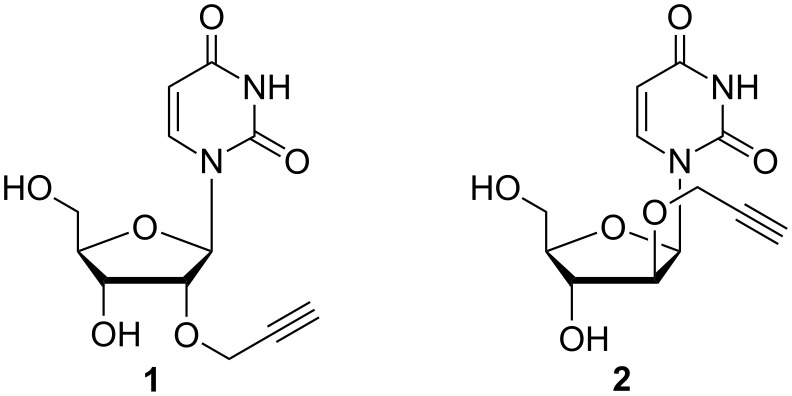
2’-Propargylated nucleosides as “clickable” DNA/RNA building blocks with ribo (**1**) and arabino (**2**) configuration.

To develop fluorescently labelled oligonucleotides that undergo energy transfer reactions [17] we recently applied 2’-propargyl-modified uridine **1** as DNA building block (Scheme 1) [15,18–19]. A simple look on the three-dimensional structure of double-helical DNA elucidates that the positioning of the fluorophores in the major groove may be improved by inversion of the configuration at the 2’-position of the anchor nucleoside sugar. In fact, arabino nucleic acids are an important class of antisense oligonucleotides [20] since their first report [21]. The orientation of the 2’-OH group in the arabino configuration towards the major groove yields hybrids with RNA that show a slightly lower thermal stability compared to DNA/RNA hybrids. In order to evaluate this structural influence for our fluorescently labelled oligonucleotides, we developed and synthesized the 2’-propargyl-modified arabino-configured uridine analog **2**, incorporated it into DNA by automated phosphoramidite chemistry, “clicked” it to a variety of our recently established, photostable cyanine-styryl dyes and probed the fluorescence and energy transfer properties by determination of quantum yields and emission color contrasts.

## Results and Discussion

**Scheme 2:**
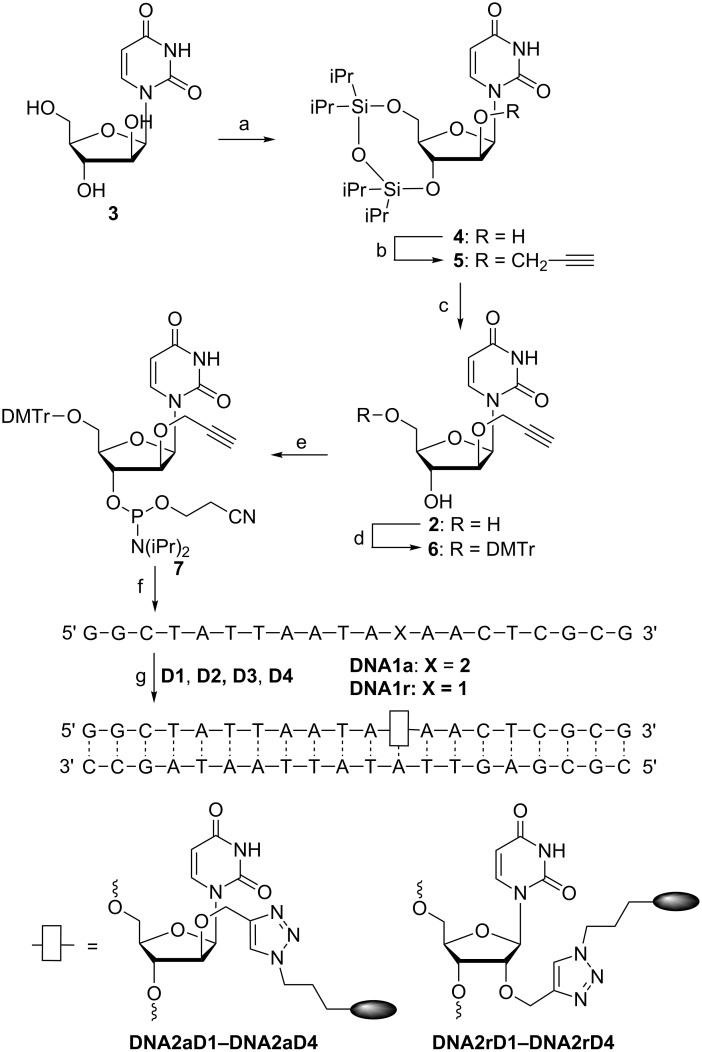
Synthesis of phosphoramidite **7** and modified DNA. a) TIPDSiCl_2_, pyridine, 2 h at 0 °C, 16 h at rt, 89%; b) 1. NaH, THF, 0 °C, 15 min, 2. propargyl bromide, rt, 18 h, 65%; c) TBAF, THF, rt, 5 min, 99%; d) DMTr-Cl, pyridine, rt, 5 h, 99%; e) 2-cyanoethyl-*N,N*-diisopropylchlorophosphoramidite, (iPr)_2_NEt, CH_2_Cl_2_, rt, 3 h, 95%; f) automated DNA synthesis; g) **D1**–**D4**, sodium ascorbate, TBTA, (CH_3_CN)_4_CuPF_6_, H_2_O/DMSO/*t*-BuOH 3:3:1, 1.5 h, 60 °C; annealing with counterstrand for 10 min at 90 °C and slow cooling to rt. For structures of **D1**–**D4** see Scheme 3.

The synthesis of the phosphoramidite **7** (Scheme 2) was straightforward and includes mainly protecting group chemistry since it starts with the commercially available arabino-configured uridine analog **3**. The 3’- and 5’-hydroxy functions of nucleoside **3** were selectively protected by the Markiewicz silyl ether [22]. The central step of the whole synthetic procedure was the alkylation of the 2’-OH function of nucleoside **4** by propargylic bromide which worked in 65% yield in the presence of NaH as base. After removal of the silyl protecting group from nucleoside **5**, the 5’-position of nucleoside **2** was again protected by 4,4’-dimethoxytrityl chloride (DMTr-Cl) and, finally, the 3’-position of nucleoside **6** was phosphitylated. Remarkably, the overall yield of phosphoramidite **7** with the optimized conditions over the described five steps is 54%. Automated DNA synthesis with **7** as building block required a slightly extended coupling time of 10 min. The phosphoramidite for the “clickable” nucleoside **1** is commercially available. After preparation, the detritylated oligonucleotides **DNA1a** (“a” = arabino) and **DNA1r** (“r” = ribo) were cleaved from the resin and deprotected with conc. NH_4_OH at 45 °C for 16 h. The lyophilized oligonucleotides were reacted with the azide-modified dyes **D1**–**D4** in the presence of Cu(I) and TBTA, as mentioned above. The reaction was performed in H_2_O/DMSO/*t-*BuOH 3:3:1 and was completed after 1.5 h at 60 °C. The modified oligonucleotides were purified by ethanol precipitation in the presence of EDTA to remove copper ions and subsequently by semi-preparative HPLC. Finally, the modified oligonucleotides were identified by MALDI–TOF mass spectrometry (see Supporting Information File 1) and annealed with the corresponding unmodified counterstrand.

The four fluorophores **D1** [23], a blue emitter excitable at 389 nm, **D2** [24], **D3** [19], and **D4** [24], all green emitters excitable at 450–460 nm, that were “clicked” to the oligonucleotides **DNA1a** and **DNA1r** belong to our recently established class of cyanine-styryl dyes that show a unique combination of optical properties [25], including suitable brightness and fluorescence quantum yields, large Stokes’ shifts compared to conventionally applied Cy3 and Cy5, and most importantly, excellent photostabilities. **D1**–**D4** were representatively chosen since they will serve as energy donors in the energy transfer-based DNA systems (vide infra). The corresponding dye azides were synthesized as previously described [19,23–24]. The modified double strands (ds) **DNA2aD1** to **DNA2aD4** were compared with their structural counterpart among the duplexes **DNA2rD1** to **DNA2rD4** with respect to their optical properties (UV–vis absorption and fluorescence, see Supporting Information File 1), fluorescence quantum yields Φ_F_ and melting temperatures *T*_m_ (Table 1). The reference duplexes of **DNA1a** and **DNA1r** annealed with the unmodified complementary strand showed *T*_m_ values of 61.0 °C and 62.0 °C, respectively. This small difference tracks well with the general observation that arabino-configured nucleic acids in general show lower stabilities than the ribo-configured ones. With the attached dyes, the arabino-modified duplexes show a smaller stabilization effect by the dyes than the corresponding ribo-modified duplexes. The stabilization of ds**DNA2a** ranges only from 0.7 °C for **D1** to 3.2 °C for **D3** and **D4**, whereas the stabilizing effects for ds**DNA2r** are more diverse, ranging from 2.0 °C for **D1** to 3.7 °C for **D3**. Obviously, the dye interactions with double-stranded DNA do slightly depend on the type of dye. In **D1** and **D2**, the pyridinium part is connected to the rest of the dye by its 4-position, in **D3** and **D4** via its 2-position. The latter connectivity has a larger stabilizing influence on the **DNA2a** double strands. The fluorescence quantum yields of ds**DNA2a** are all higher than the corresponding ones of ds**DNA2r**. Especially in case of **D2** Φ_F_ could be significantly improved from 27% to 45%, and in case of **D4** from 9% to 16%. This is remarkable and clearly shows that the arabino-configured nucleoside **2** provides the structurally optimized anchor for fluorescent dye interactions with the DNA. Obviously, placing the dyes into the major groove led them find a better orientation than in the minor groove, with respect to the DNA helix with enhanced fluorescence intensities.

**Table 1:** Melting temperatures (*T*_m_) and fluorescence quantum yields (Φ_F_) of singly modified **DNA2aD1–DNA2rD4**.

dye	λ_exc_ (nm)	**DNA2a…** *T*_m_ [°C]	Φ_F_	**DNA2r…** *T*_m_ [°C]	Φ_F_

**…D1**	389	61.9	0.096^a^	65.7	0.052^a^
**…D2**	462	61.7	0.452^b^	64.0	0.266^b^
**…D3**	450	64.2	0.136^c^	65.1	0.122^c^
**…D4**	462	64.2	0.156^d^	65.2	0.087^d^

^a^λ_em_ = 404–800 nm; ^b^λ_em_ = 477–800 nm; ^c^λ_em_ = 480–800 nm; ^d^λ_em_ = 473–800 nm.

**Scheme 3:**
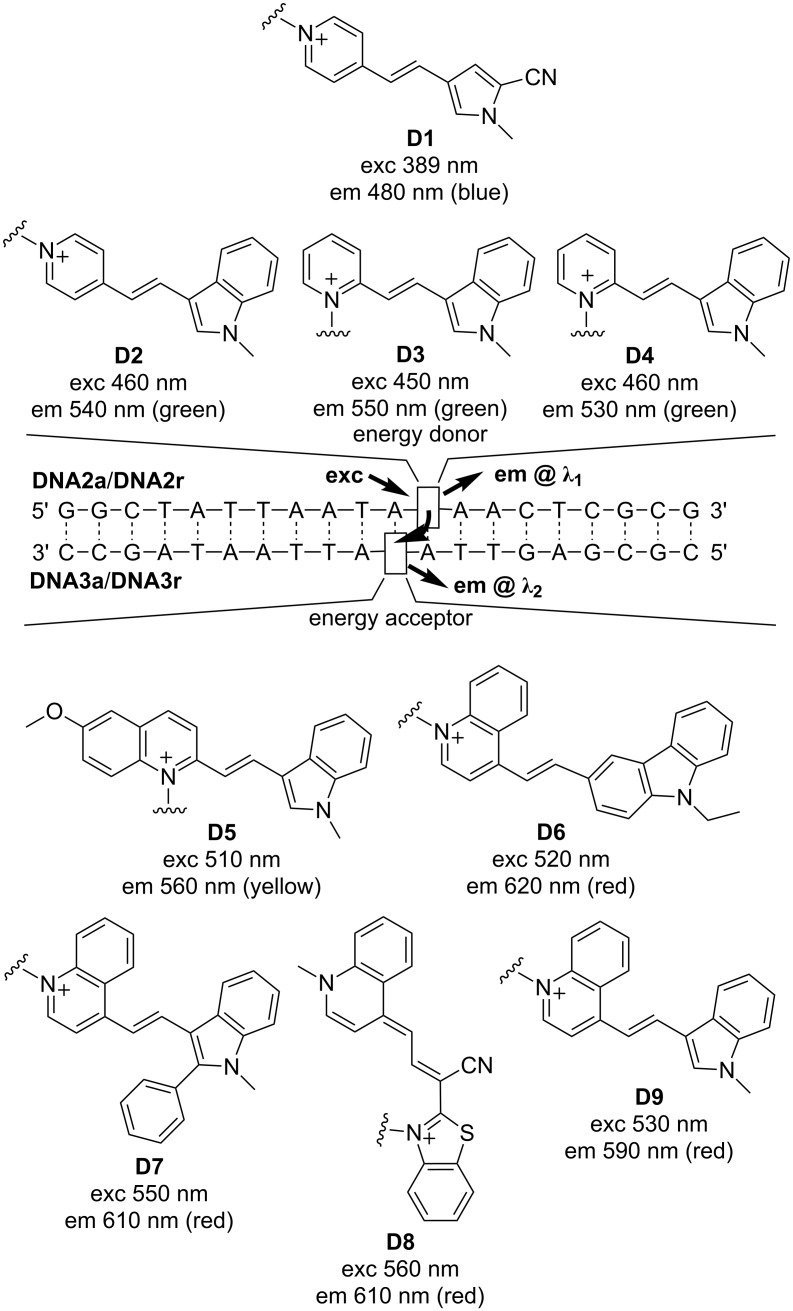
Structures of donor dyes **D1**–**D4** as modifications of **DNA2a** and **DNA2r** and structures of acceptor dyes **D5**–**D8** as modifications of **DNA3a** and **DNA3r** yielding energy transfer-based nucleic acid probes.

The dyes **D1**–**D4** as energy donors were combined with dyes **D5**–**D9** as energy acceptors (Scheme 3). This approach follows our concept of “DNA/RNA traffic lights” [17,19,25] that are energy transfer-based nucleic acid probes that can be used in molecular beacons [26], especially for vesicular microRNA imaging in living cancer cells [27], and for siRNA transport imaging [28]. Donor and acceptor dyes are combined in an interstrand and diagonal orientation to promote best possible energy transfer. In particular, we combined each of the eight oligonucleotides **DNA2a** and **DNA2r** modified with **D1**–**D4** with each of the ten oligonucleotides **DNA3a** and **DNA3r** modified with **D5** [29], **D6** [25], **D7** [30], **D8** [19] and **D9** [29]. In detail, the blue emitting dye **D1** combines to a blue–yellow fluorophore pair (**D1**/**D5**) and to blue–red emitting pairs (**D1**/**D6–D1**/**D9**). The green emitting dyes **D2**–**D4** result all in green–red emitting pairs (**D2**/**D7**–**D4**/**D9**). The combination of dyes **D2**–**D4** with **D5** and **D6** is not meaningful for this concept since the fluorescence of the donors and absorption of the acceptors show broad spectral overlays and therefore selective excitation is not possible.

For each of the previously described dye combinations, we probed all four combinations of arabino- and ribo-configured donor strands (**DNA2a** and **DNA2r**) with acceptor strands (**DNA3a** and **DNA3r**). This array of doubly modified DNA duplexes was screened by their emission color contrast C = *I*_Ac_/*I*_Do_ (fluorescence intensity near maximum of acceptor divided by fluorescence intensity near maximum of donor) and for the fluorescence quantum yield Φ_F_ in the range of the acceptor emission (Table 2). The comparison with our previously applied approach to link both donor and acceptor dyes at ribo-configured nucleoside **1** (in **DNA2r** and **DNA3r**) revealed that the emission color contrasts are not improved when they are both anchored at the arabino-configured nucleoside **2** (in **DNA2a** and **DNA3a**). There are only very few excemptions; especially the combination **DNA2aD4**–**DNA3aD8** yields a red-to-green contrast of 83 compared to 45 in case of the ribo-configured **DNA2rD4**–**DNA3rD8**. Additionally, the fluorescence quantum yield was improved from 39% to 53%. The dye combination **D2**/**D8** nicely demonstrates the effect of the arabino-configured attachment because the corresponding duplexes show all enhanced quantum yields whereas the pure ribo configuration in **DNA2rD2**–**DNA3rD8** quenches its fluorescence significantly. The latter example (**DNA2rD2**–**DNA3rD8**) shows an altered absorbance of the two dyes which gives an important photophysical insight. An efficient energy transfer between two dyes requires the selective excitation of an uncoupled donor in the proximity to an uncoupled acceptor. Hyper-/hypochromicity and/or shifted absorbance of the dyes indicate excitonic (ground state) interactions between the dyes which interfere with the energy transfer between them [31].

**Table 2:** Fluorescence intensity ratios (color contrast) C = *I*_Ac_/*I*_Do_ and fluorescence quantum yields Φ_F_ of energy transfer pairs between dyes **D1–D4** in **DNA2a** and **DNA2r** and dyes **D5–D9** in **DNA3a and DNA3r**. The abbreviations a and r are listed in the order according to the duplex formation between **DNA2** (first letter) with **DNA3** (second letter), for instance a–r means **DNA2a**–**DNA3r**.

Do→ Ac↓		**DNA2a** and **DNA2r**							
**DNA3a** and **DNA3r**		**D1**		**D2**		**D3**		**D4**	
		C	Φ_F_	C	Φ_F_	C	Φ_F_	C	Φ_F_

**D5**	**a**–**a** **a**–**r** **r**–**a** **r**–**r**	35 198 129 70	0.146^a^ 0.606 0.224 0.217	– – – –	– – – –	– – – –	– – – –	– – – –	– – – –
**D6**	**a**–**a** **a**–**r** **r**–**a** **r**–**r**	15 48 11 40	0.148^b^ 0.227 0.127 0.206	– – – –	– – – –	– – – –	– – – –	– – – –-	– – – –
**D7**	**a**–**a** **a**–**r** **r**–**a** **r**–**r**	44 85 46 93	0.273^c^ 0.357 0.213 0.340	20 36 10 60	0.237^d^ 0.245 0.210 0.312	41 177 82 136	0.198^d^ 0.319 0.218 0.218	69 39 108 153	0.212^d^ 0.214 0.268 0.229
**D8**	**a**–**a** **a**–**r** **r**–**a** **r**–**r**	109 80 215 87	0.606^c^ 0.576 0.719 0.545	20 12 15 3	0.466^e^ 0.427 0.378 0.078	41 43 77 40	0.528^f^ 0.564 0.549 0.388	83 48 86 45	0.672^e^ 0.592 0.534 0.366
**D9**	**a**–**a** **a**–**r** **r**–**a** **r**–**r**	60 58 215 69	0.307^g^ 0.306 0.240 0.245	11 23 7 25	0.222^h^ 0.245 0.132 0.226	9 62 30 59	0.148^h^ 0.285 0.237 0.244	28 27 34 38	0.220^h^ 0.258 0.184 0.206

^a^λ_exc_ = 389 nm, λ_em_ = 515–800 nm; ^b^λ_exc_ = 389 nm, λ_em_ = 525–800 nm; ^c^λ_exc_ = 389 nm, λ_em_ = 550–800 nm; ^d^λ_exc_ = 435 nm, λ_em_ = 550–800 nm; ^e^λ_exc_ = 430 nm, λ_em_ = 550–800 nm; ^f^λ_exc_ = 430 nm, λ_em_ = 540–800 nm; ^g^λ_exc_ = 389 nm, λ_em_ = 530–800 nm; ^h^λ_exc_ = 423 nm, λ_em_ = 550–800 nm.

**Figure 1:**
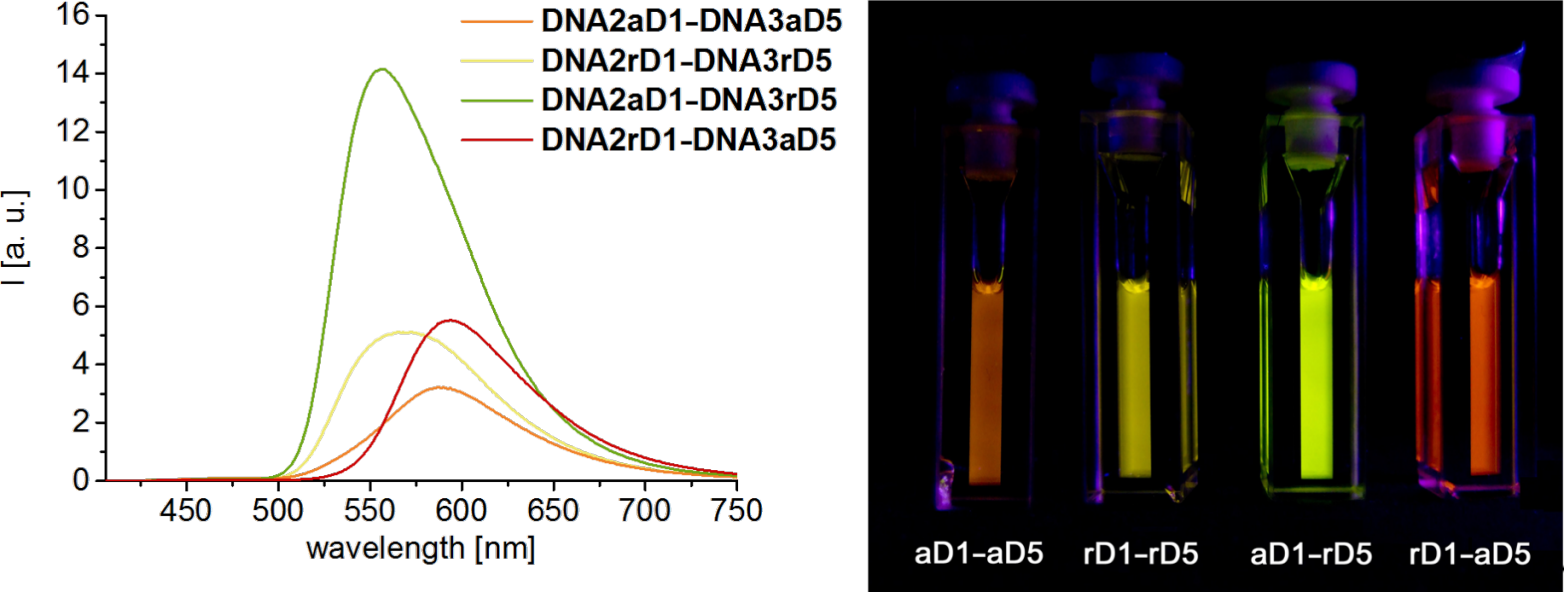
Representative demonstration of the fluorescence readout differences between the four arabino/ribo combinations of **D1** (donor) and **D5** (acceptor). Left: Fluorescence of **DNA2a/rD1**–**DNA3a/rD5**; 2.5 μM DNA in 50 mM Na-P_i_ buffer, 250 mM NaCl, pH 7, λ_exc_ = 391 nm. Right: Corresponding image of cuvettes excited by a handheld UV lamp.

Among the tested combinations, there are some remarkable examples in this array in which mixed energy transfer duplexes, meaning the combination of donor dyes linked to arabino-configured nucleosides (**DNA2a**) with acceptor dyes attached to ribo-configured nucleosides (**DNA3r**) and vice versa (**DNA2r** with **DNA3a**) yield significantly enhanced emission color contrasts. As a representative example, the fluorescence color readout for the combinations of **D1** with **D5** (Figure 1) ranges from green (**DNA2aD1**–**DNA3rD5**) to orange/red (**DNA2rD1**–**DNA3aD5**). Especially, the combination **DNA2aD1**–**DNA3rD5** revealed a yellow-to-blue contrast of 198 and a quantum yield of 61%. For the blue–red emitting dye combinations the highest red-to-blue contrast of 215 and the highest quantum yield of 71% is achieved in **DNA2rD1**–**DNA3aD8**. Finally, among the broadest array of green–red fluorophore pairs there are a few remarkable duplexes with superior energy transfer parameters. Representatively, it is noteworthy that the combination **DNA2aD3**–**DNA3rD7** gives a red-to-green contrast of 177 (and a quantum yield of 32%), and the combination **DNA2rD4**–**DNA2aD8** shows a quantum yield of 53% (and a red-to-green contrast of 86).

**Figure 2:**
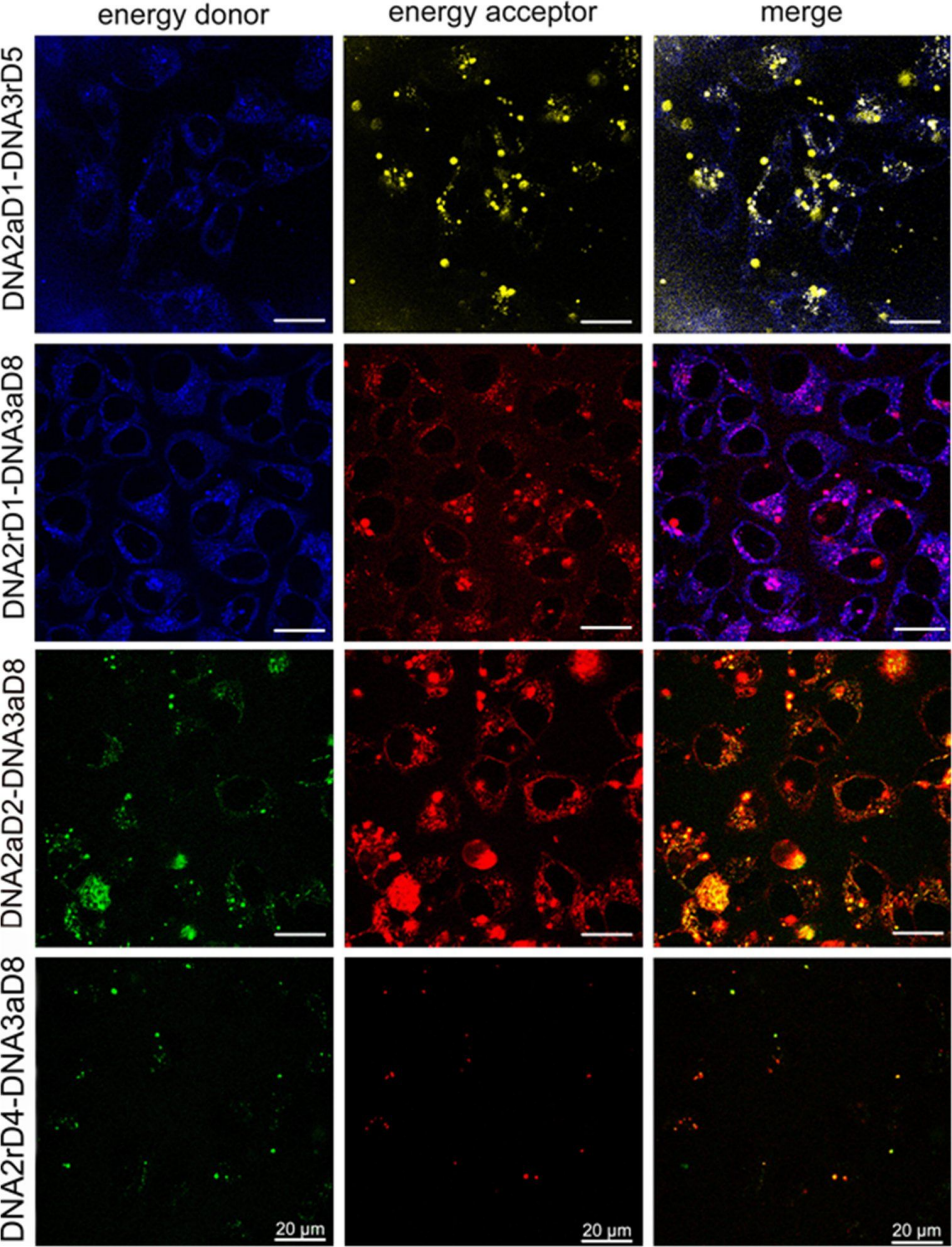
Confocal microscopy of HeLa cells after transfection with **DNA2aD1**–**DNA3rD5** (row 1), **DNA2rD1**–**DNA3aD8** (row 2), **DNA2aD2–DNA3aD8** (row 3) and **DNA2rD4**–**DNA3aD8** (row 4). The visualization was performed using a Leica TCS-SPE (DMi8) inverted microscope with an ACS APO 63×/1.30 oil objective. For **DNA2aD1**–**DNA3rD5** λ_exc_ = 405 nm (UV laser), λ_em_ = 435–470 nm (blue) and 575–750 nm (yellow), for **DNA2rD1**–**DNA3aD8** λ_exc_ = 405 nm (UV laser), λ_em_ = 415–550 nm (blue) and 575–750 nm (red), for **DNA2aD2–DNA3aD8** λ_exc_ = 488 nm (argon ion laser), λ_em_ = 490–550 nm (green) and 550–675 nm (red), for **DNA2rD4**–**DNA3aD8** λ_exc_ = 488 nm (argon ion laser), λ_em_ = 490–550 nm (green) and 675–800 nm (red), scale bar = 20 µm.

In order to test the functionality of the respective dyes as FRET pairs in DNA duplexes for imaging in cells, four representative duplexes, **DNA2aD1**–**DNA3rD5**, **DNA2rD1–DNA3aD8**, **DNA2aD2–DNA3aD8** and **DNA2rD4**–**DNA3aD8**, were tested in HeLa cells. 5 × 10^4^ HeLa cells were transiently transfected with 15 pmol of the above mentioned DNA duplexes and Screenfect^®^, for 24 hours at a concentration, which was not toxic for the cells (see cytotoxicity test in Supporting Information File 1), and imaged by confocal fluorescent microscopy using the excitation wavelength of the energy donor (**D1**, λ_exc_ = 405 nm, **D2**, λ_exc_ = 488 nm, **D4**, λ_exc_ = 488 nm). To analyze the energy transfer to the energy acceptor the fluorescence of the energy donor (**D1**, λ_em_ = 435–470 nm (blue), **D2**, λ_em_ = 490–550 nm (green), Figure 2, left column) and the respective energy acceptor dye (**D5**, λ_em_ = 575–750 nm (yellow), **D8**, λ_em_ = 575–750 nm (red), Figure 2, middle column) was detected. In comparison to non-transfected control cells specific fluorescent staining could be observed in the perinuclear region, indicating that all dyes tested were endocytosed by the cells. The DNA duplexes preferentially accumulated in endosomal/lysosomal vesicles. The fluorescence of the energy donors, **D1**, **D2** and **D4** (Figure 2, left column), as well as the fluorescence of the energy acceptors, **D5** and **D8** (Figure 2, middle column), could be detected showing that fluorescence energy was transferred from the donor to the acceptor in the respective FRET pairs in the endosomal vesicles. This suggested that the DNA duplexes were still intact after transfection into cells.

## Conclusion

The phosphoramidite **7** bearing the arabino-configured analog of uridine **2** that is additionally propargylated at the 2’-position was easily synthesized from commercially available nucleoside precursor **3** in 54% yield over five steps. The fluorescence quantum yields of oligonucleotides that were postsynthetically modified by the blue emitting dye **D1** and the green-emitting dyes **D2**–**D4** were improved due to the arabino-configured anchor **2** in comparison to the conventional ribo-configured uridine **1**. This rather small structural difference allows the attached fluorophores to point into the major groove. Thereby optimized dye–DNA orientations result in higher fluorescence quantum yields of these single dye modifications. The modified oligonucleotides with dyes **D1**–**D4** were applied as energy donors together with the correspondingly modified oligonucleotides bearing the acceptor dyes **D5**–**D9**. All dyes belong to our recently established class of cyanine-styryl dyes that show excellent photostabilities. The two-by-two combinations of these dyes give energy transfer pairs with blue-to-yellow, blue-to-red and green-to-red emission color changes. For these dye combinations, we probed all four combinations of arabino- and ribo-configured donor strands with arabino- and ribo-configured acceptor strands, and screened this array of doubly modified DNA duplexes by their emission color contrast C and the fluorescence quantum yield Φ_F_. This screening revealed that the combination of donor and acceptor dyes does not necessarily yield better optical properties if they are both linked to the arabino-configured nucleoside **2** (compared to the linkage to the ribo-configured nucleoside **1**). However, there are some remarkable examples in this array of duplexes with mixed combinations, that means donor dyes linked to the arabino-configured nucleoside **2** with acceptor dyes linked to the ribo-configured nucleoside **1**, and vice versa, that showed significantly improved emission color contrasts and/or fluorescence quantum yields. Thereby, improved fluorescent nucleic acid probes were elucidated that are suitable not only for nucleic acid imaging of living cells but additionally allow a two-color readout.

## Experimental

**Materials and methods.** Chemicals and dry solvents were purchased from Aldrich, ABCR, and VWR and were used without further purification unless otherwise stated. Unmodified oligonucleotides were purchased from Metabion. TLC was performed on Fluka silica gel 60 F254 coated aluminum foil. FAB mass spectra were measured by the analytical facilitites of the Institute of Organic Chemistry (KIT) using a Finnigan MAT95 in positive ionization mode. NMR spectra were recorded on a Bruker B-ACS-60, Bruker Avance DRX 400 and a Bruker Avance DRX 500 spectrometer in deuterated solvents (^1^H at 300, 400 or 500 MHz, ^13^C at 75, 100 or 125 MHz). Chemical shifts are given in ppm relative to TMS. IR spectra were recorded by the analytical facility of the Institute of Organic Chemistry (KIT) on a Bruker IFS88 spectrometer.

Optical-spectroscopic measurements were recorded in NaP_i_-buffer solution (10 mM, pH 7) with 250 mM NaCl in quartz glass cuvettes (10 mm). Absorption spectra were recorded with a Varian Cary 100 spectrometer equipped with a 6 × 6 cell changer unit at 20 °C. Fluorescence was measured with a Jobin–Yvon Fluoromax 3 fluorimeter with a step width of 1 nm and an integration time of 0.2 s. All spectra were recorded at 20 °C and are corrected for Raman emission from the buffer solution. Quantum yields were determined with Quantaurus QY C11347 of Hamamatsu.

**DNA2aD1** to **DNA2aD4**, **DNA2rD1** to **DNA2rD4**, **DNA3aD5** to **DNA3aD9** and **DNA3rD5** to **DNA3rD9** were purified using a reversed-phase Supelcosil™ LC-C18 column (250 × 10 mm, 5 µm) on a Shimadzu HPLC system (autosampler, SIL-10AD, pump LC-10AT, controller SCL-10A, diode array detector SPD-M10A). Purification was confirmed by MS (MALDI–TOF) on a Biflex-IV spectrometer from Bruker Daltonics in the linear negative mode (matrix: 1:9 mixture of diammonium hydrogencitrate (100 g/L) and a saturated 3-hydroxypicolinic acid solution (10 g/L in 50% acetonitrile in water)). DNA concentrations were measured by their absorbance in water at 260 nm on a ND-1000 spectrometer from NanoDrop in the nucleic acid mode.

**Synthesis of 4.** 1-Deoxy-1-(uracil-1-yl)-β-D-arabinofuranose (**3**, 1.00 g; 4.10 mmol) was dried under reduced pressure for 1 h and was then dissolved in dry pyridine (5 mL). The reaction mixture was cooled to 0 °C and TIPDSiCl_2_ (1.44 mL, 4.51 mmol) was slowly added. After 2 h, the reaction mixture was warmed to room temperature and stirred overnight. The solvent was removed under reduced pressure and the remaining solid was purified by flash chromatography (SiO_2_, 0 → 50% EtOAc in CH_2_Cl_2_). 1.78 g (3.66 mmol, 89%) of **4** as a colorless solid were obtained. Spectral data were in accordance with the literature [32].

**Synthesis of 5.** Under argon atmosphere **4** (1.02 g, 2.10 mmol) was dissolved in dry THF (20 mL) and cooled to 0 °C with an ice bath. Then NaH (0.168 g, 4.20 mmol of 60% dispersion in mineral oil) was added and the reaction mixture was stirred for 15 min at 0 °C. The reaction mixture was warmed to room temperature and propargyl bromide (0.94 mL, 1.25 g, 8.40 mmol) was added slowly within 30 minutes. The reaction was stirred for 18 h at room temperature and quenched by adding distilled water (10 mL). The mixture was extracted with ethyl acetate (two times 100 mL). The combined organic layers were washed with saturated NaHCO_3_ solution and then dried over Na_2_SO_4_. The solvent was removed under reduced pressure and the residue was purified by column chromatography (SiO_2_, 0–40% EtOAc in hexane) to obtain **5** (0.716 g, 1.37 mmol, 65%) as a colorless foam. *R*_f_ 0.40 (hexane/EtOAc 1:1); ^1^H NMR (400 MHz, CDCl_3_) δ 8.48 (s, 1H, NH), 7.62 (d, *J* = 8.1 Hz, 1H, H-6), 6.22 (d, *J* = 6.0 Hz, 1H, H-1’), 5.63 (m, 1H, OH-3’), 5.69 (d, *J* = 8.2 Hz, 1H, H-5), 4.37 (dd, *J* = 7.7 Hz, 6.1 Hz, 1H, H-1’), 4.28–4.19 (m, 3H, OCH_2_, H-3’), 4.07 (dd, *J* = 13.2 Hz, 2.4 Hz, 1H, H-5_a_’), 4.00 (dd, *J* = 13.2 Hz, 2.9 Hz, 1H, H-5_b_’), 3.73 (dt, *J* = 8.6 Hz, 2.6 Hz, 1H, H-4’), 2.42 (t, J = 2.4 Hz, 1H, CH), 1.11–0.95 (m, 28H, 8× CH_3_ & 4× CH) ppm; ^13^C NMR (75 MHz, CDCl_3_) δ 160.1 (C-4), 150.4 (C-2), 140.9 (C-6), 101.9 (C-5), 82.2 (C-1’), 82.2 (C-4’), 80.4 (C-2’), 78.9 (C-CH), 75.5 (CH), 72.4 (C-3’), 60.5 (C-5’), 59.5 (OCH_2_), 17.6–17.0 (8 CH_3_), 13.6–12.5 (4 CH) ppm; FAB–MS *m*/*z* (%): 525.2 (65) [M + H]^+^; FAB–HRMS FAB *m*/*z*: [M + H]^+^calcd for C_24_H_41_N_2_O_7_Si_2_^+^, 525.2447; found, 525.2447.

**Synthesis of 2.** Under an Ar atmosphere **5** (0.687 g, 1.31 mmol) was dissolved in dry THF (17 mL) 1 M tetrabutylammonium fluoride in THF (3.28 mL, 3.28 mmol) was added. The reaction was stirred for 5 min at room temperature. The reaction solution was directly poured onto a short silica plug and eluted with CH_2_Cl_2_/MeOH 5:1. The solvent was removed under reduced pressure and the crude product was purified by column chromatography (SiO_2_, CH_2_Cl_2_/MeOH 10:1) to afford **2** (0.366 g, 1.30 mmol, 99%) as a colorless foam. *R*_f_ 0.24 (CH_2_Cl_2_/MeOH 9:1); ^1^H NMR (300 MHz, DMSO-*d*_6_) δ 11.33 (s, 1H, NH), 7.65 (d, *J* = 8.1 Hz, 1H, H-6), 6.13 (m, 1H, H-1’), 5.63 (m, 1H, OH-3’) 5.60 (d, *J* = 8.1 Hz, 1H, H-5), 5.02 (m, 1H, OH-5’), 4.16 (d, *J* = 2.4 Hz, 2H, OCH_2_), 4.13–4.02 (m, 2H, H-2’, H-3’), 3.72–3.49 (m, 3H, H-4’, 2 H-5’), 3.44 (t, *J* = 2.3 Hz, 1H, CH) ppm; ^13^C NMR (126 MHz, DMSO-*d*_6_) δ 163.1 (C-4), 150.4 (C-2), 141.9 (C-6), 100.6 (C-5), 83.2 (C-1’), 82.8 (C-4’), 82.4 (C-2’), 79.5 (C-CH), 77.6 (CH), 72.5 (C-3’), 59.9 (C-5’), 57.5 (OCH_2_) ppm; FAB–MS *m*/*z* (%): 242.3 (100) [M]^+^ − CH_2_CCH (propargyl).

**Synthesis of 6.** Under an inert gas atmosphere **2** (0.543 g, 1.26 mmol) was dissolved in dry pyridine (14 mL), 4,4’-dimethoxytrityl chloride (0.510 g, 1.51 mmol) was added in one portion and the reaction mixture was then stirred for 5 h at room temperature. The reaction was quenched by adding MeOH (5 mL) and the solvents were removed under reduced pressure. The residue was dissolved in EtOAc (20 mL). The organic layer was washed with 1 M aqueous NaHCO_3_ solution (3 times 20 mL), dried over Na_2_SO_4_, and the solvent was removed under reduced pressure. The crude product was purified by column chromatography (SiO_2_, CH_2_Cl_2_/MeOH 99:1 + 0.1% NEt_3_) to afford **6** (0.729 g, 1.25 mmol, 99%) as a colorless foam. *R*_f_ 0.13 (CH_2_Cl_2_/MeOH 50:1); ^1^H NMR (400 MHz, CDCl_3_) δ 7.75 (d, *J* = 8.1 Hz, 1H, H-6), 7.44–7.22 (m, 9H, DMTr), 6.88–6.81 (m, 4H, DMTr), 6.28 (d, *J* = 5.8 Hz, 1H, H-1’), 5.43 (d, *J* = 8.2 Hz, 1H, H-5), 4.39 (dd, *J* = 7.0 Hz, 5.7 Hz, 1H, H-2’), 4.28–4.10 (m, 3H, OCH_2_, H-3’), 3.89 (dt, *J* = 7.1 Hz, 3.6 Hz, 1H, H-3’), 3.52 (dd, *J* = 10.8 Hz, 3.6 Hz, 1H H-5_a_’), 3.46 (dd, *J* = 10.8 Hz, 3.8 Hz, 1H H-5_b_’), 2.49 (t, *J* = 2.4 Hz, 1H, CH) ppm; ^13^C NMR (101 MHz, DMSO-*d*_6_) δ 163.09, 158.81, 150.39, 144.52, 141.7, 135.5, 135.5, 130.3, 130.2, 128.3, 128.2, 127.3, 113.4, 101.7, 87.0, 83.6, 83.3, 81.0, 79.2, 77.4, 75.8, 74.0, 61.6, 59.0, 55.4 ppm; FAB–MS *m*/*z* (%): 585.1 (68) [M + H]^+^ ; FAB–HRMS *m*/*z*: [M + H]^+^calcd for C_33_H_33_N_2_O_8_^+^, 585.2231; found, 585.2231.

**Synthesis of 7.** In a round bottom flask **6** (0.196 g, 0.34 mmol) was dried overnight under vacuum and then dissolved in dry CH_2_Cl_2_ (5 mL) under an Ar atmosphere. *N,N*-Diisopropylethylamine (175 µL, 1.01 mmol) and 2-cyanoethyl *N,N*-diisopropylchlorophosphoramidite (119 µL, 0.50 mmol) were added. The reaction mixture was stirred for 3 h at room temperature and then directly purified by column chromatography (SiO_2_, CH_2_Cl_2_/acetone 5:1 + 0.1% NEt_3_). **6** (0.253 g, 0.32 mmol, 95%) was obtained as a colorless foam. *R*_f_ 0.56 (CH_2_Cl_2_/acetone 5:1); APCI–MS *m*/*z* (%): 785.6 (70) [M + H]^+^.

**Preparation, purification and characterization of DNA.** All oligonucleotides were synthesized on an Expedite 8909 Synthesizer from Applied Biosystems (ABI) using standard phosphoramidite chemistry. Reagents and CPG (1 µmol) were purchased from Proligo. The commercially available ribo-configured 2’-*O*-propargyluridine was purchased from ChemGenes. For the arabino-configured building block **7** a slightly extended coupling time of 10 minutes was used. After preparation, the trityl-off oligonucleotides were cleaved from the resin and deprotected with conc. NH_4_OH at 45 °C for 16 h.

**Click reaction with modified oligonucleotides.** To the lyophilized alkyne-modified DNA sample were added water (100 µL), sodium ascorbate (25 µL of 0.4 M in water), tris[(1-benzyl-1*H*-1,2,3-triazol-4-yl)methyl]amine (34 µL of 0.1 M in DMSO/*t-*BuOH 3:1), dye azide (114 µL of 0.01 M in DMSO/*t-*BuOH 3:1) and tetrakis(acetonitrile) copper(I) hexafluorophosphate (17 µL of 0.1 M in DMSO/*t-*BuOH 3:1). The reaction mixture was kept at 60 °C for 1.5 h. After cooling to room temperature, the DNA was precipitated by adding Na_2_EDTA (150 µL of 0.05 M in water), sodium acetate (450 µL of 0.3 M in water) and ethanol (10 mL, 100%) and stored at −32 °C for 16 h. After centrifugation, the supernatant was removed and the residue washed two times with cold ethanol (2 mL, 80%). The dried DNA pellet was then further purified via HPLC as further described in Supporting Information File 1.

**Cell experiments and confocal fluorescence microsopy.** Human cervix carcinoma cells (HeLa cells) were cultured in Dulbecco’s modified Eagle medium (DMEM) supplemented with 10% fetal calf serum and 1% penicillin/streptomycin at 37 °C in a 5% CO_2_ atmosphere. 24 h before transfection 5 × 10^4^ HeLa cells per well were seeded in an 8-well chamber slide (µ Slide 8 well ibiTreat, IBIDI, Martinsried, Germany) in 200 µL of media. For the transfection 15 pmol of the respective DNA duplexes were diluted in ScreenFect^®^A dilution buffer (Incella, Eggenstein-Leopoldshafen, Germany) to a final volume of 9 µL. 12 µL of a 1:10 dilution of ScreenFect^®^A in dilution buffer were added to the diluted DNA and rapidly mixed. A subsequent incubation time of 20 min at room temperature allowed the formation of lipoplexes (liposome–DNA complexes). The transfection mixture was then added to the cells. The cells were incubated for 24 h with the respective transfection mixture at 37 °C in a 5% CO_2_ atmosphere. The visualization of the DNA duplexes was performed by confocal laser scanning microscopy using a Leica TCS SPE (DMi8) inverted microscope with an ACS APO 63×/1.30 oil objective. Fluorophores were excited using an UV laser (405 nm) for duplexes **DNA2aD1**–**DNA3rD5** and **DNA2rD1**–**DNA3aD8** and an argon ion laser (488 nm) for duplexes **DNA2aD2**–**DNA3aD8** and **DNA2rD4**–**DNA2aD8**. The emission detection bandwidths were at 435–470 nm (blue) and 575–750 nm (yellow) for **DNA2aD1**–**DNA3rD5**, 415–550 nm (blue) and 575–750 nm (red) for **DNA2rD1**–**DNA3aD8**, 490–550 nm (green) and 550–675 nm (red) for **DNA2aD2**–**DNA3aD8**, 490–550 nm (green) and 675–800 nm (red) for **DNA2rD4**–**DNA2aD8**. Using the acquisition software Leica Application Suite (LAS) X 2.0.1.14392, the picture ratio was adjusted to 1024 × 1024 pixels 8 bit depth.

## Supporting Information

File 1 Additional data and spectra.
