# Supplementary material for: A postsynthetically 2’-“clickable” uridine with arabino configuration and its application for fluorescent labeling and imaging of DNA
Source: Beilstein J Org Chem. 2017 Jan 20;13:127–37. doi: 10.3762/bjoc.13.16 (PMC5302004; doi:10.3762/bjoc.13.16)
Supplement: File 1 — Additional data and spectra. [file Beilstein_J_Org_Chem-13-127-s001.pdf]

Supporting Information

for

A postsynthetically 2'-“clickable” uridine with arabino configuration and its application for fluorescent labeling and imaging of DNA

Heidi-Kristin Walter[1], Bettina Olshausen[2], Ute Schepers[2] and Hans-Achim Wagenknecht*[1]

Address: [1]Institute of Organic Chemistry, Karlsruhe Institute of Technology (KIT), Fritz-Haber-Weg 6, 76131 Karlsruhe, Germany and [2]Institute of Toxicology and Genetics, Karlsruhe Institute of Technology (KIT), H.-v.-Helmholtz-Platz 1, 76344 Eggenstein-Leopoldshafen, Germany

Email: Hans-Achim Wagenknecht - Wagenknecht@kit.edu

*Corresponding author

Additional data and spectra

Contents

1. Images of NMR and MS analyses S2

2. HPLC purification of modified DNA and images of HPLC analytics S9

3. Images of MALDI-TOF MS analyses of modified DNA S18

4. Optical spectra for single-and double stranded modified DNA S28

5. Melting temperature of modified DNA double strands S34

6. Additional cell images S35

7. Cytotoxicity S36

1. Images of NMR and MS analyses

Compound 5

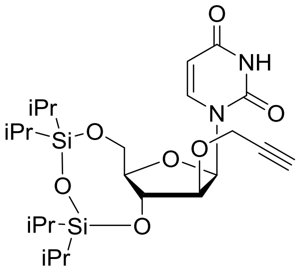

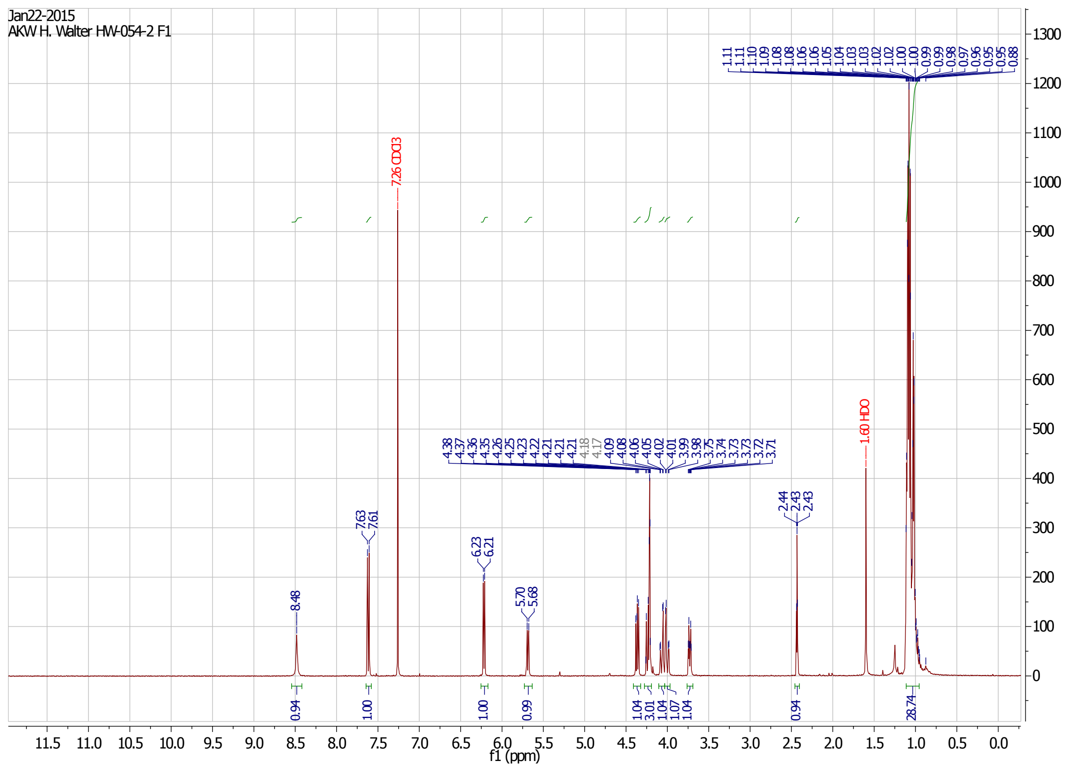

Figure S1: ^{1}H NMR spectrum of **5**.

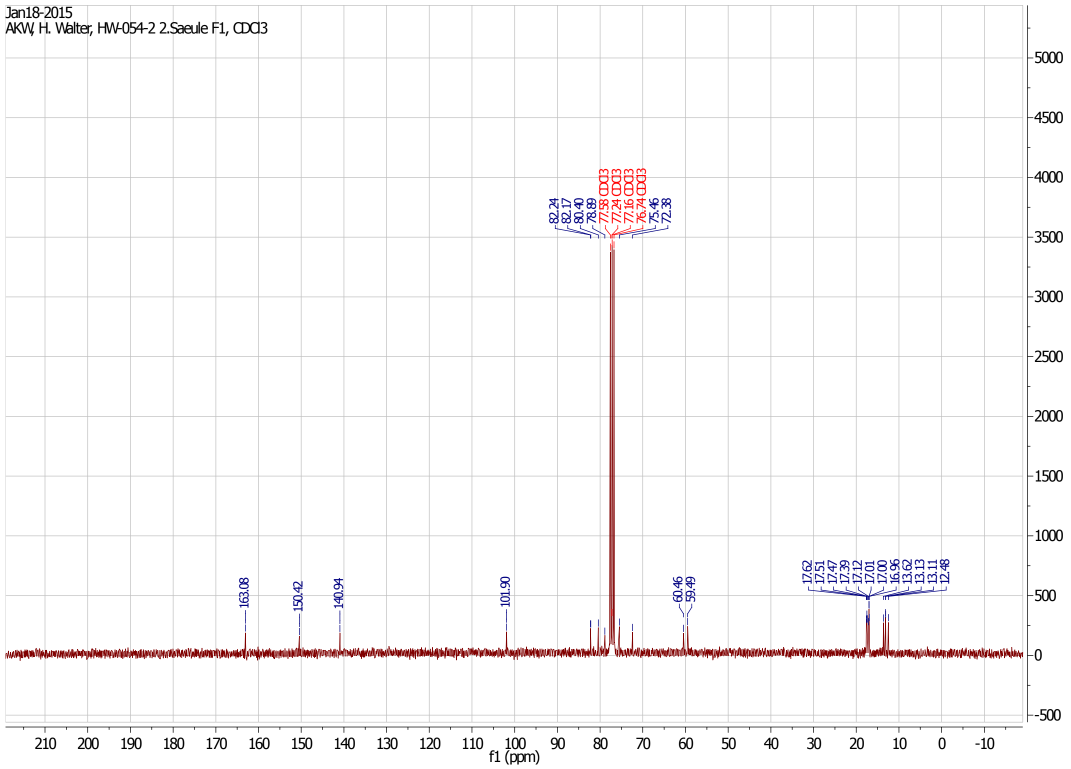

Figure S2: ^{13}C NMR spectrum of **5**.

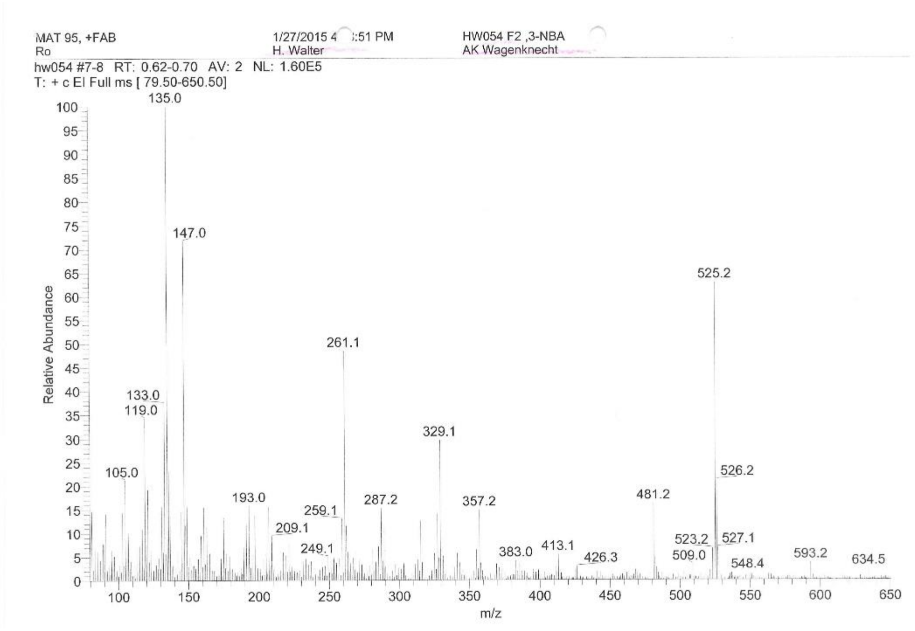

Figure S3: MS (FAB) analysis of **5**.

1/27/2015 4. :14 PM File recalibrated by CM[illegible]

hw054-c8#18 RT: 1.50
T: + c EI Full ms [79.57-650.57]
m/z= 524.9702-525.4872

m/z	Intensity	Relative	Theo. Mass	Delta (mmu)	Composition
525.2447	317962.0	100.00	525.2447	0.05	$C_{24}H_{41}O_7N_2{}^{28}Si_2$

Figure S4: HR-MS (FAB) analysis of **5**.

Compound 2

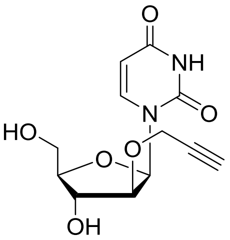

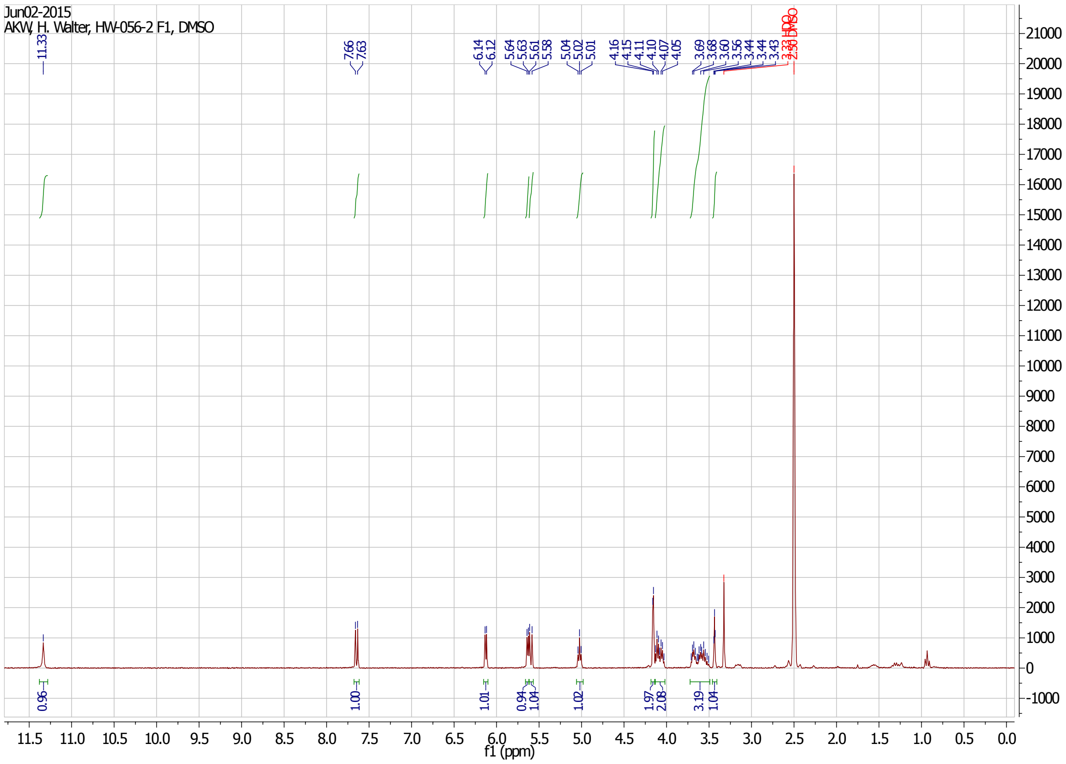

Figure S5: ^{1}H NMR spectrum of **2**.

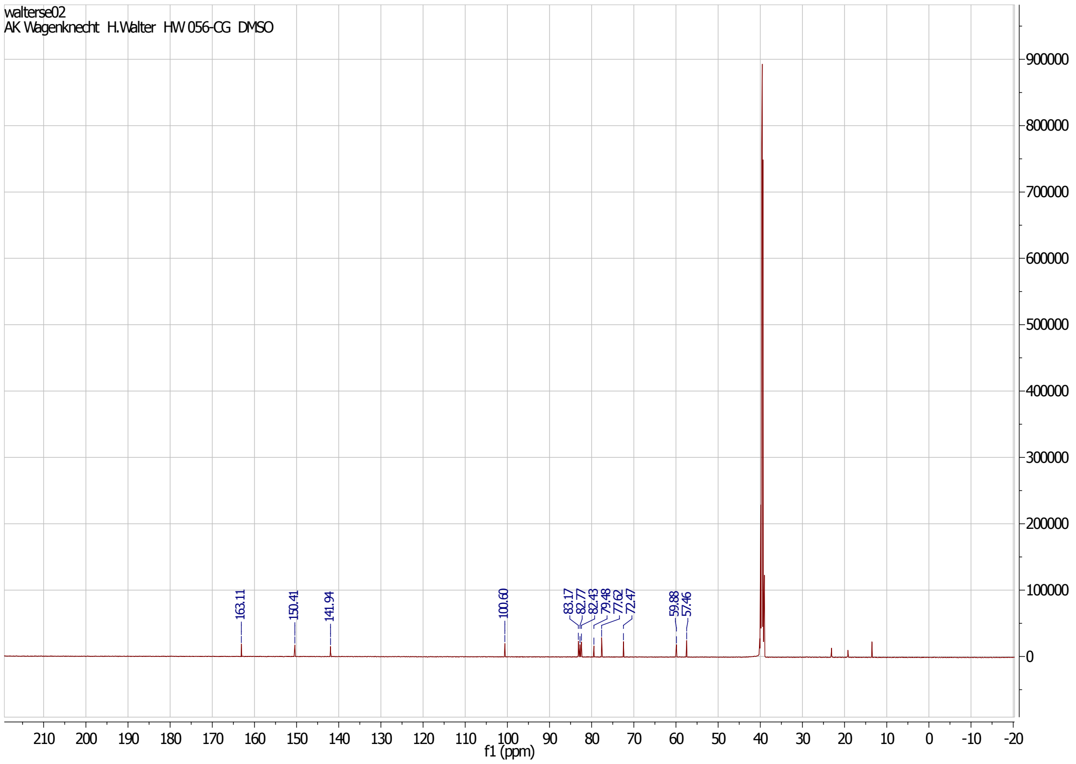

Figure S6: ^{13}C NMR spectrum of **2**.

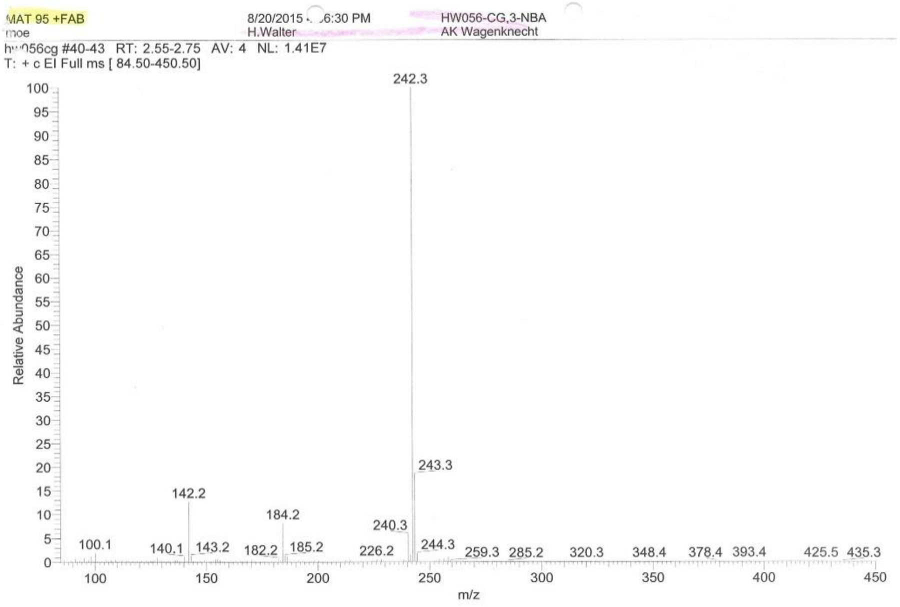

Figure S7: MS (FAB) analysis of **2**.

Compound 6

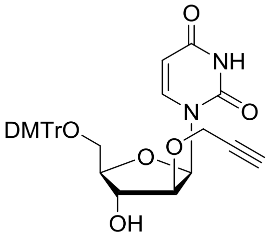

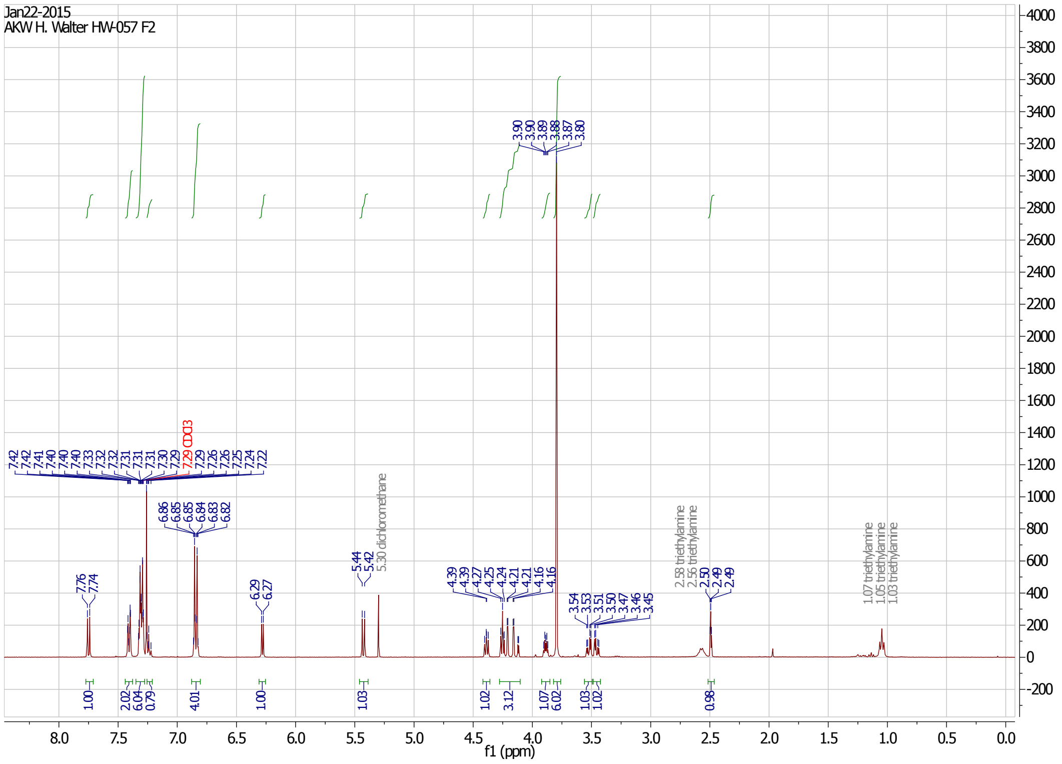

Figure S8: ^{1}H NMR spectrum of **6**.

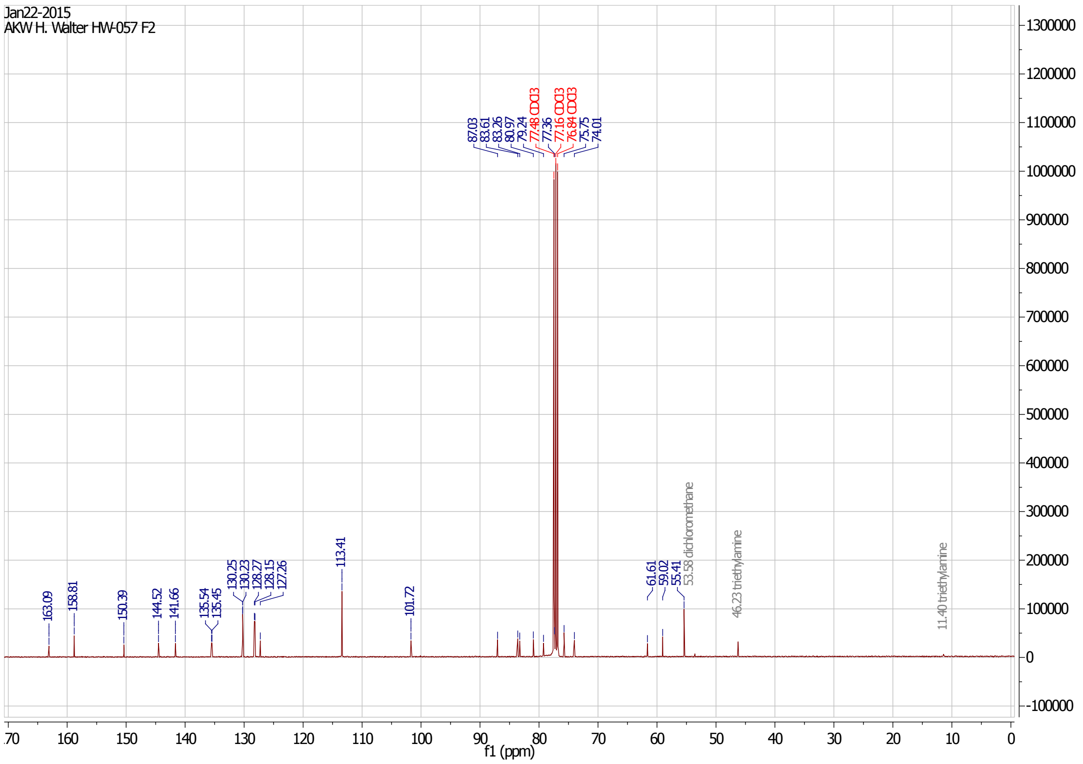

Figure S9: ^{13}C NMR spectrum of **6**.

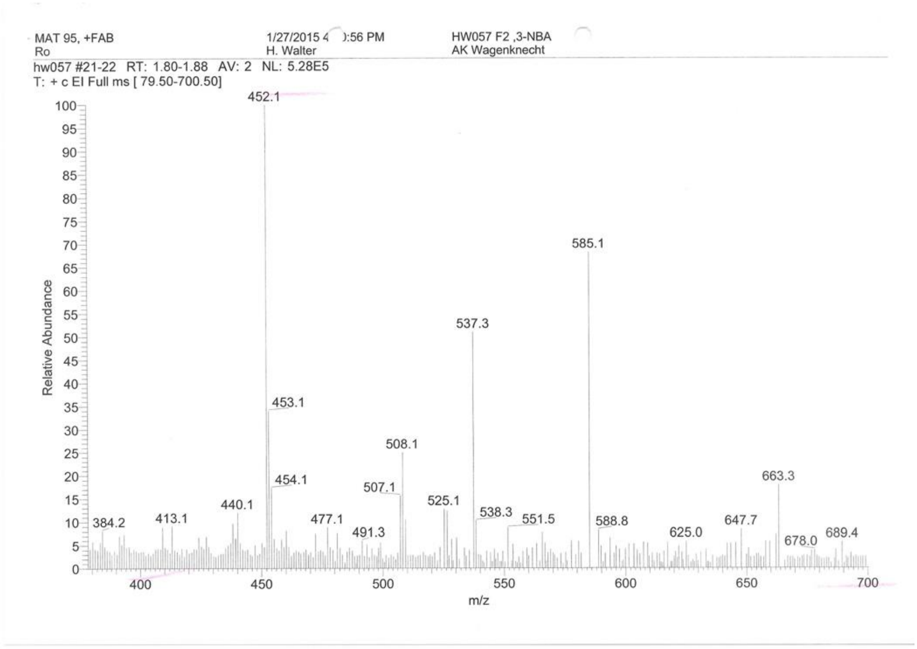

Figure S10: MS (FAB) analysis of **6**.

1/27/2015 [illegible]:54 PM File recalibrated by CM[illegible]s.

hw057-c5#19 RT: 1.63
T: + c EI Full ms [79.57-700.57]
m/z= 584.7564-585.4026

m/z	Intensity	Relative	Theo. Mass	Delta (mmu)	Composition
585.2231	109150.0	100.00	585.2231	-0.04	$C_{33} H_{33} O_8 N_2$

Figure S11: HR-MS (FAB) analysis of **6**.

Compound 7

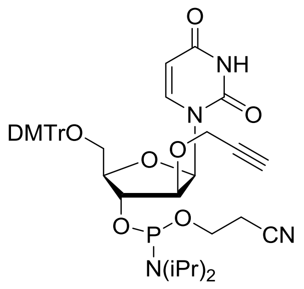

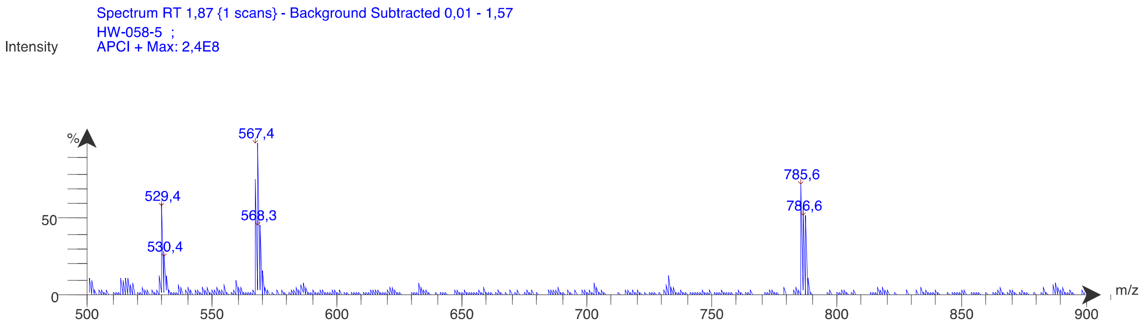

Figure S12: MS (APCI) analysis of **7**.

2. HPLC purification of modified DNA and images of HPLC analytics

The labelled DNA strands were purified via HPLC Reversed Phase Supelcosil^{TM} LC-C18 column (250 × 10 mm, 5 µm) on a Shimadzu HPLC system (autosampler, SIL-10AD, pump LC-10AT, controller SCL-10A, diode array detector SPD-M10A) using the following conditions:

eluent A:	NH_4OAc buffer (0.05 M in water, pH 6.5)
eluent B:	acetonitrile
flow rate:	2.5 mL/mL

For gradients see **Table S1:** UV–vis detection at 260 nm and the characteristic absorption wavelengths for each dye which are listed below:

**DNA2aD1/DNA2rD1**	385 nm
**DNA2aD2/DNA2rD2**	459 nm
**DNA2aD3/DNA2rD3**	444 nm
**DNA2aD4/DNA2rD4**	461 nm
**DNA3aD5/DNA3rD5**	497 nm
**DNA3aD6/DNA3rD6**	509 nm
**DNA3aD7/DNA3rD7**	542 nm
**DNA3aD8/DNA3rD8**	585 nm
**DNA3aD9/DNA3rD9**	530 nm

Table S1: HPLC gradients for semi-preparative purification of oligonucleotides **DNA2aD1**–**DNA2aD9 and DNA2rD1** – **DNA2rD9**. [a] **DNA1a/DNA1r**; [b] **DNA2aD1/DNA2rD1**; [c] **DNA3aD6/DNA3rD6**; [d] **DNA2aD2/DNA2rD2/DNA2aD3/DNA2rD3/DNA2aD4/DNA2rD4/DNA3aD5/DNA3rD5/DNA3aD8/DNA3rD8**; [e] **DNA3aD7/DNA3rD7/DNA3aD9/DNA3rD9**.

**time [min]**	**eluent B [%]**
0	0
45	8[a]/10[b]/12[c]/15[d]/17[e]
65	8[a]/10[b]/12[c]/15[d]/17[e]
66	80
75	80
76	0
85	0

Analytical HPLC of the purified DNA samples were performed with reversed phase Supelcosil^{TM} LC-C18 column (250 × 4.5 mm, 5 µm) on a Shimadzu HPLC system (autosampler, SIL-10AD, pump LC-10AT, controller SCL-10A, diode array detector SPD-M10A) using the following conditions:

eluent A: NH_4OAc buffer (0.05 M in water, pH 6.5)
eluent B: acetonitrile
flow rate: 1.0 mL/min

For gradients see **Table S2.** UV–vis detection at 260 nm and the characteristic absorption wavelengths for each dye (see chapter 0)

Table S2: HPLC-gradients for analytical determination of purified oligonucleotides **DNA1a**, **DNA1r**, **DNA2aD1–DNA2aD4**, **DNA2rD1**–**DNA2rD4**, **DNA3aD5**–**DNA2aD9** and **DNA3rD5**–**DNA3rD9**.

**time [min]**	**eluent B [%]**
0	0
45	20
60	20
61	80
70	80
71	0
75	0

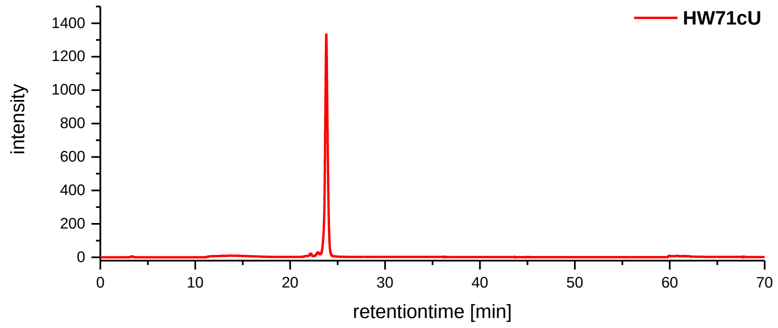

Figure S13: HPLC analysis of purified **DNA1r**.

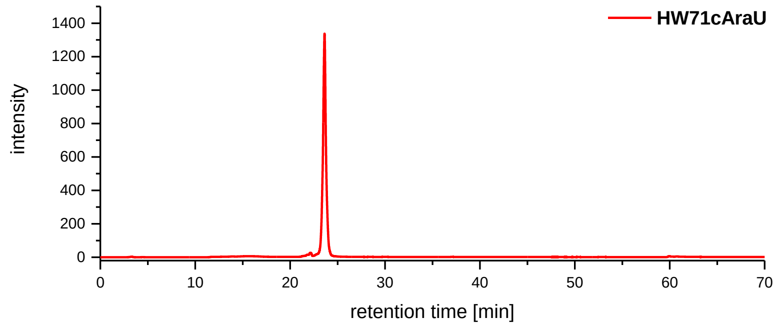

Figure S14: HPLC analysis of purified **DNA1a**.

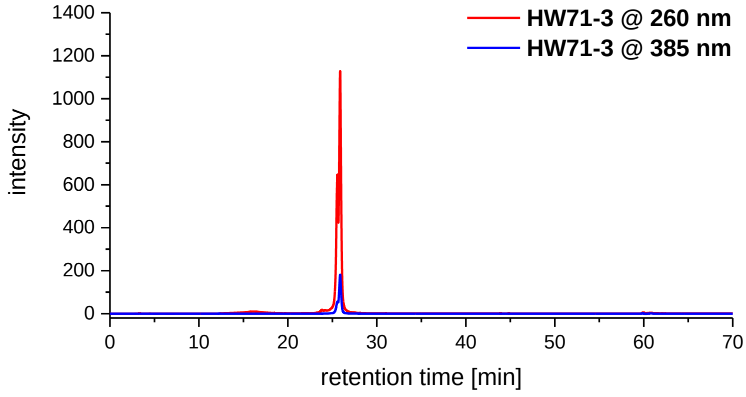

Figure S15: HPLC analysis of purified **DNA2aD1**.

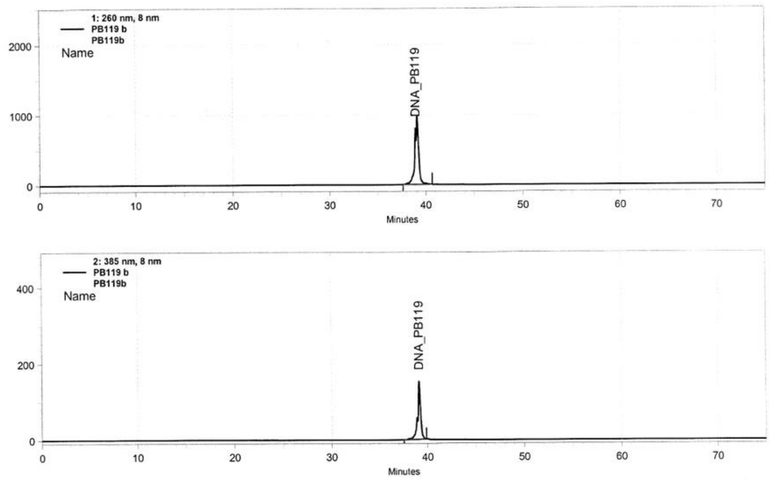

Figure S16: HPLC analysis of purified **DNA2rD1**.

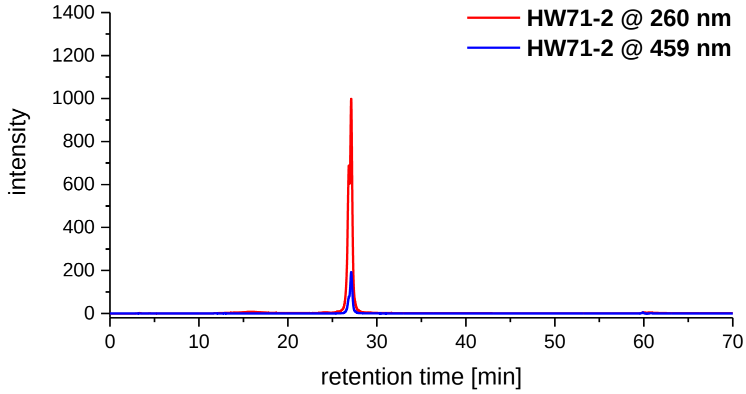

Figure S17: HPLC analysis of purified **DNA2aD2**.

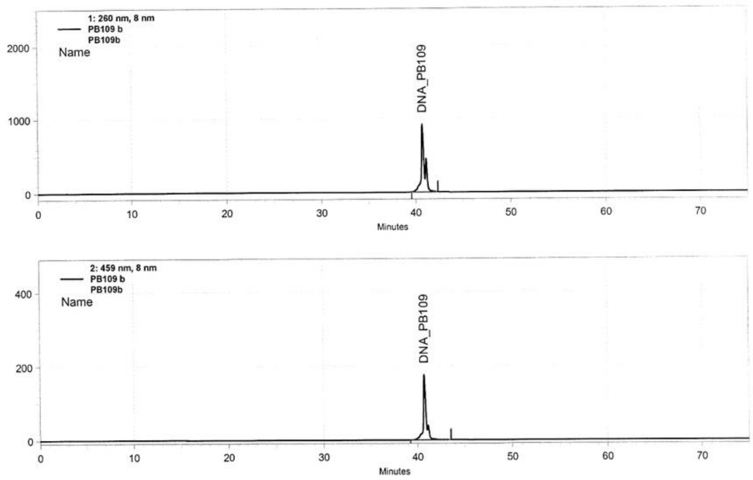

Figure S18: HPLC analysis of purified **DNA2rD2**.

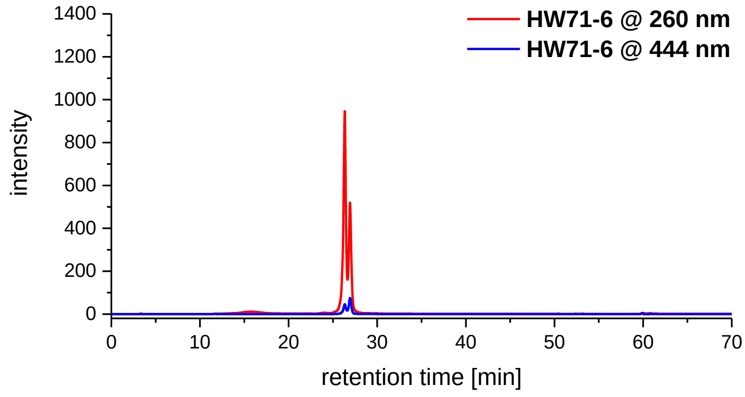

Figure S19: HPLC analysis of purified **DNA2aD3**.

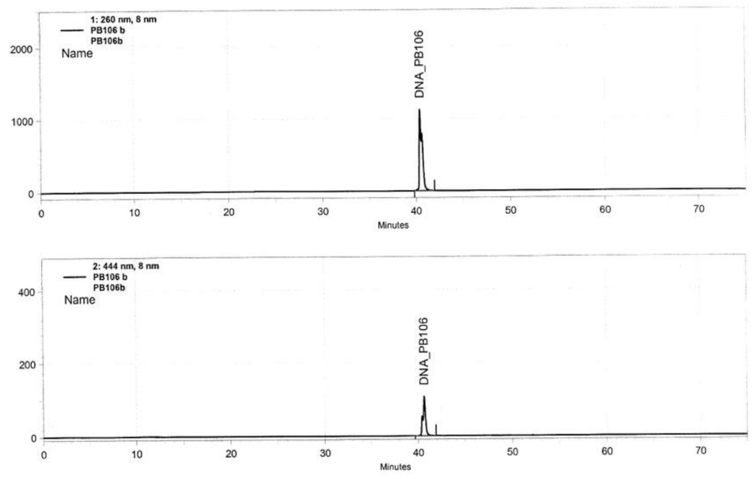

Figure S20: HPLC analysis of purified **DNA2rD3**.

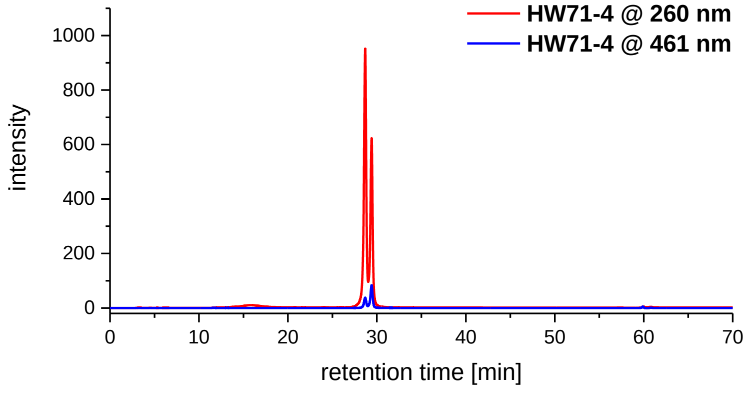

Figure S21: HPLC analysis of purified **DNA2aD4**.

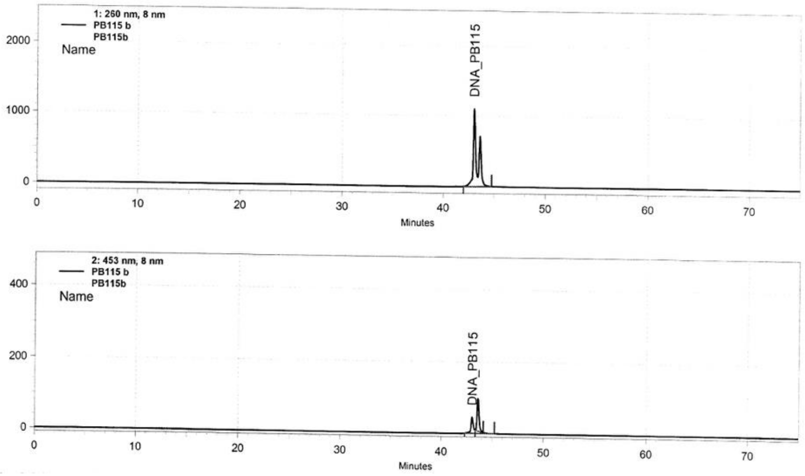

Figure S22: HPLC analysis of purified **DNA2rD4**.

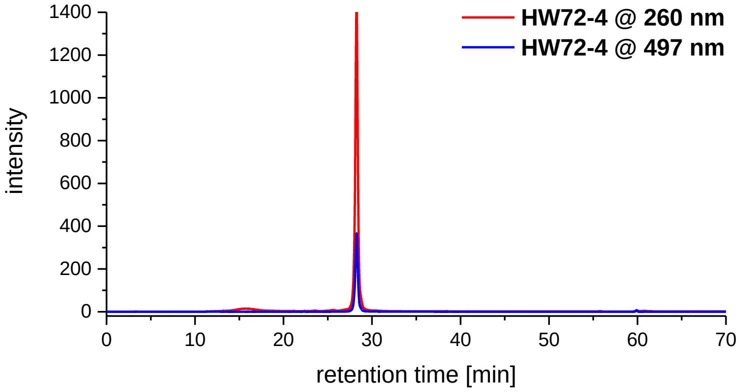

Figure S23: HPLC analysis of purified **DNA3aD5**.

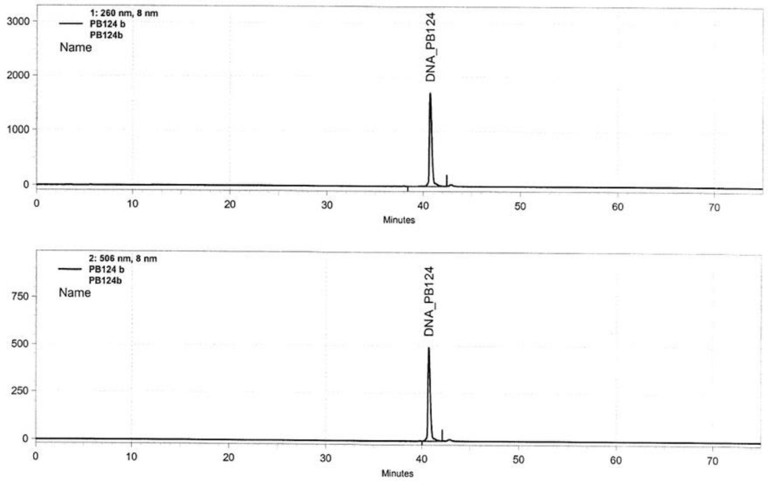

Figure S24: HPLC analysis of purified **DNA3rD5**.

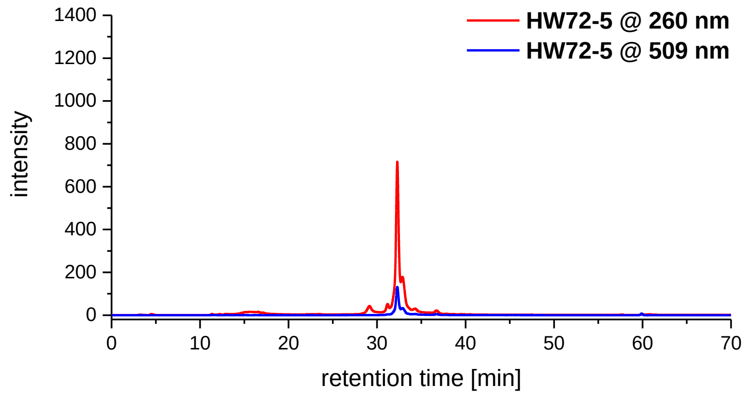

Figure S25: HPLC analyis of purified **DNA3aD6**.

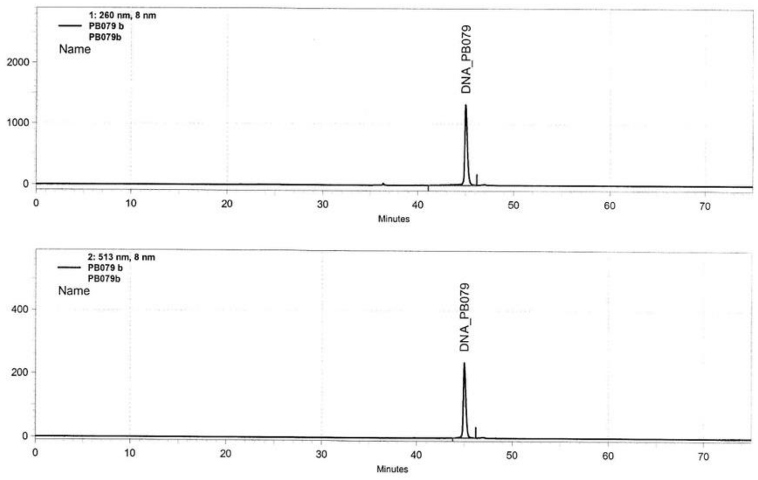

Figure S26: HPLC analysis of purified **DNA3rD6**.

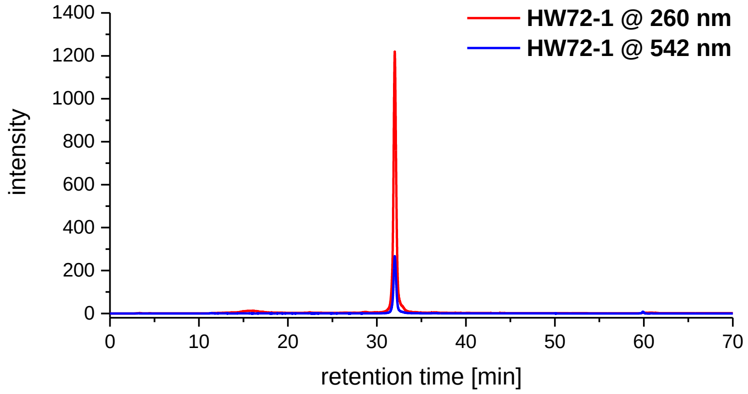

Figure S27: HPLC analysis of purified **DNA3aD7**.

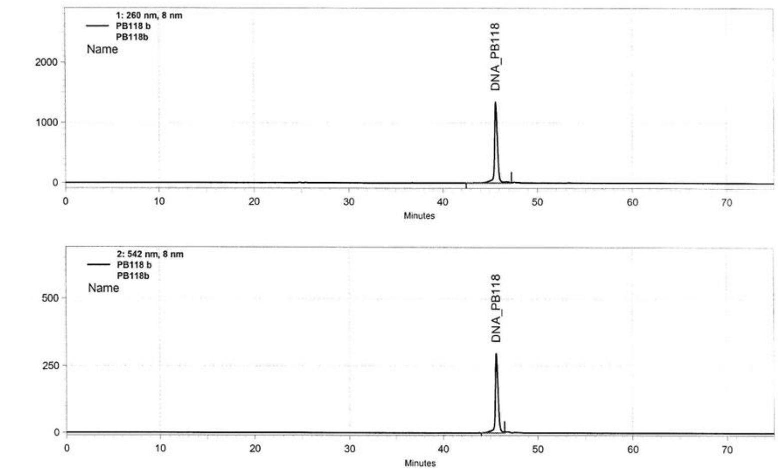

Figure S28: HPLC analysis of purified **DNA3rD7**.

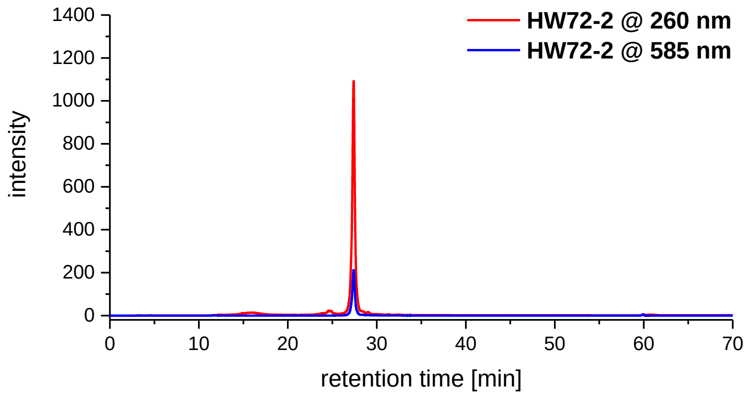

Figure S29: HPLC analysis of purified **DNA3aD8**.

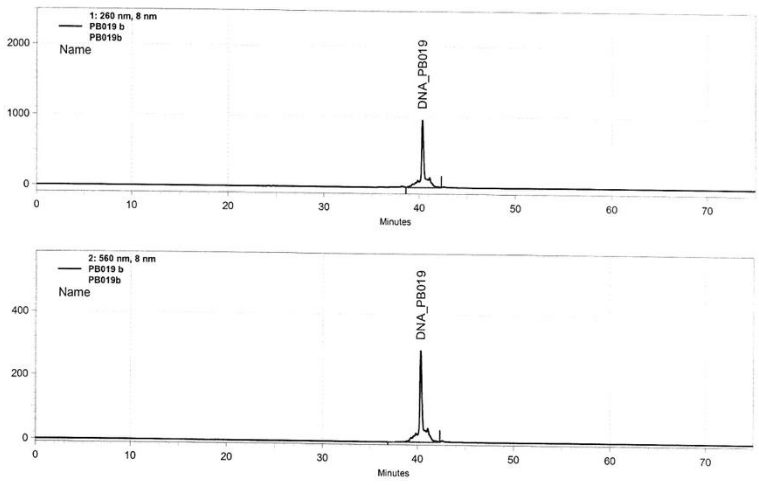

Figure S30: HPLC analysis of purified **DNA3rD8**.

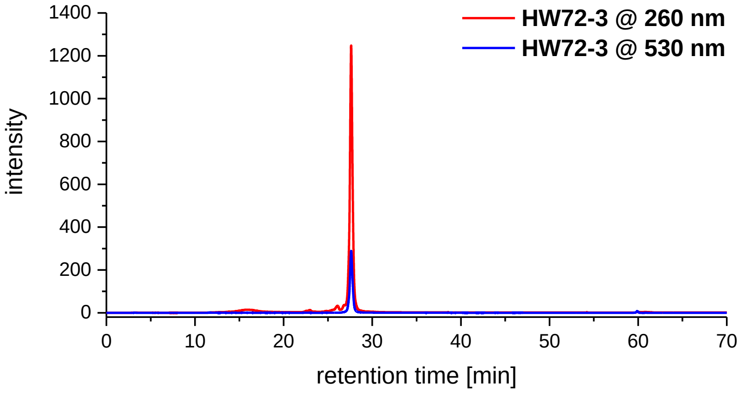

Figure S31: HPLC analysis of purified **DNA3aD9**.

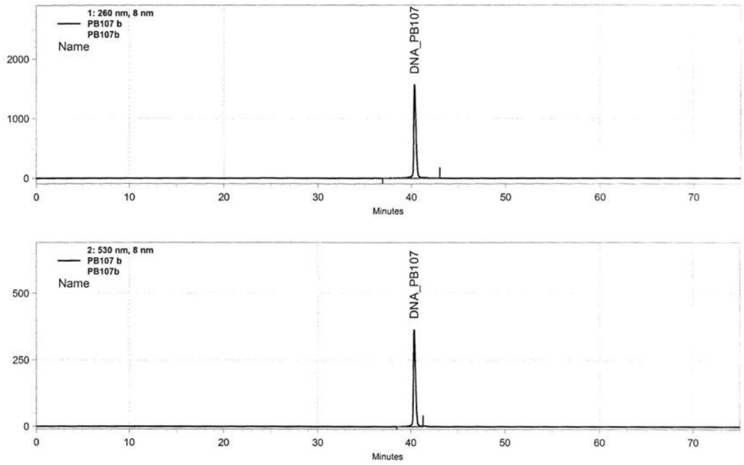

Figure S32: HPLC analysis of purified **DNA3rD9**.

3. Images of MALDI-TOF MS analyses of modified DNA

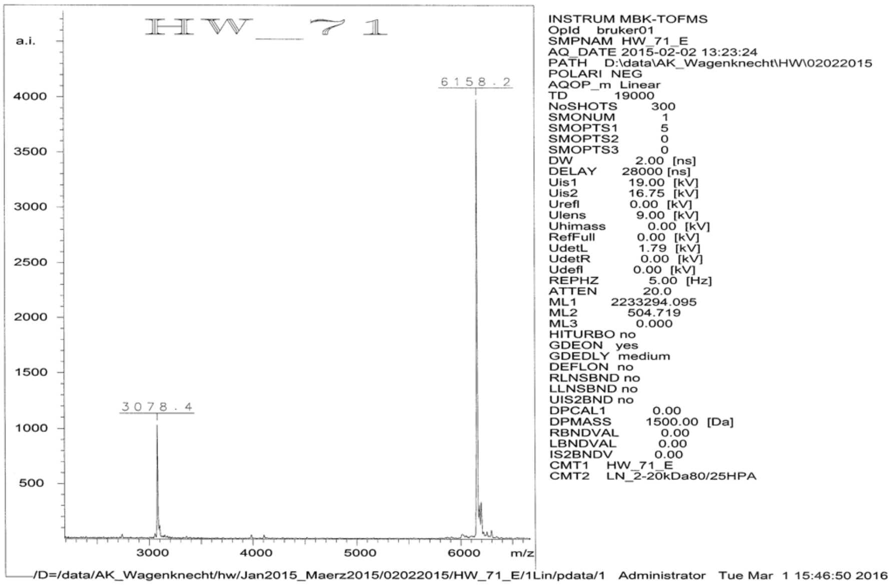

Figure S33: MALDI-TOF MS analysis of **DNA1a**; calculated: 6153.2 Da, found: 6158.2 Da.

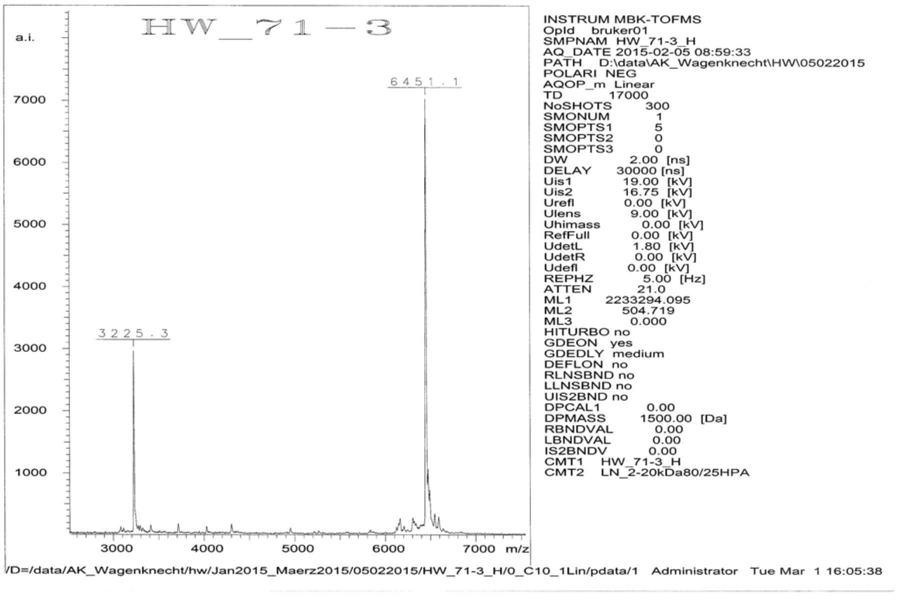

Figure S34: MALDI-TOF MS analysis of **DNA2aD1**; calculated: 6445.2 Da, found: 6451.1 Da.

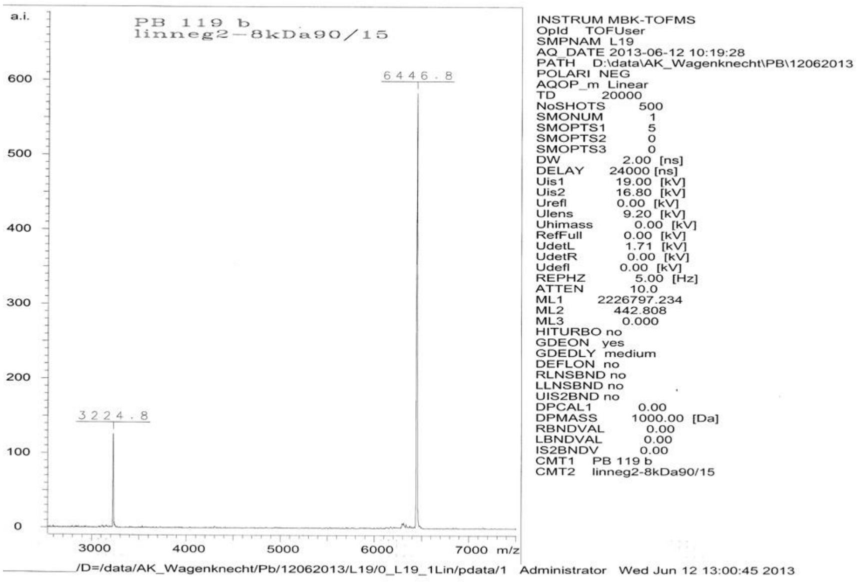

Figure S35: MALDI-TOF MS analysis of **DNA2rD1**; calculated: 6445.2 Da, found: 6446.8 Da.

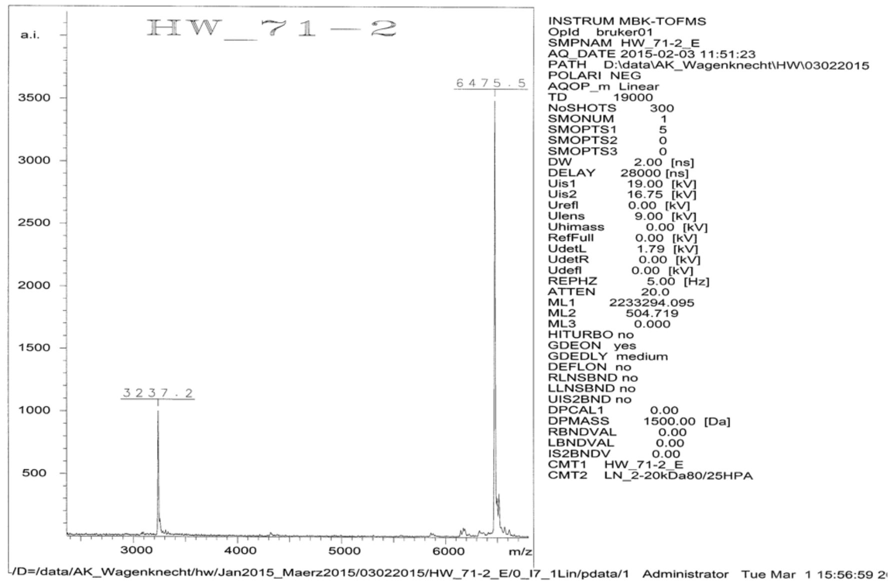

Figure S36: MALDI-TOF MS analysis of **DNA2aD2**; calculated: 6471.2 Da, found: 6475.5 Da.

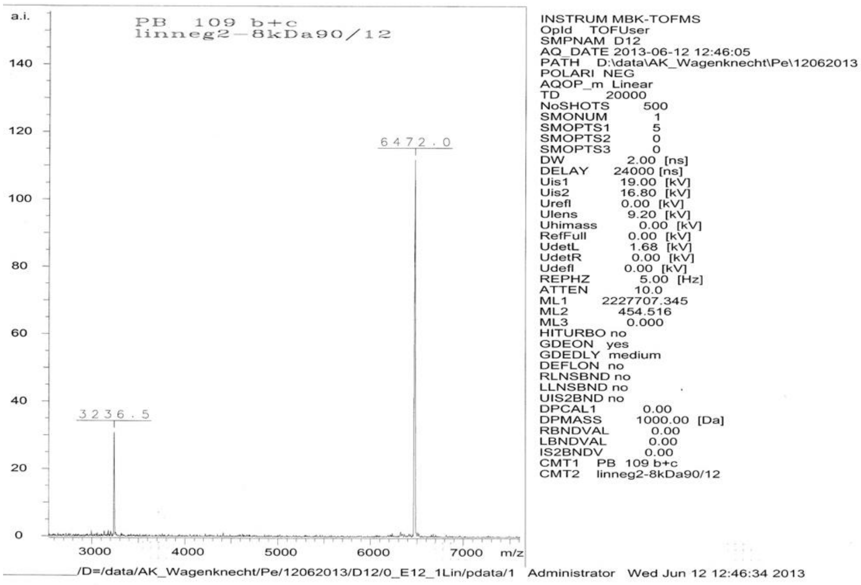

Figure S37: MALDI-TOF MS analysis of **DNA2rD2**; calculated: 6471.2 Da, found: 6472.0 Da.

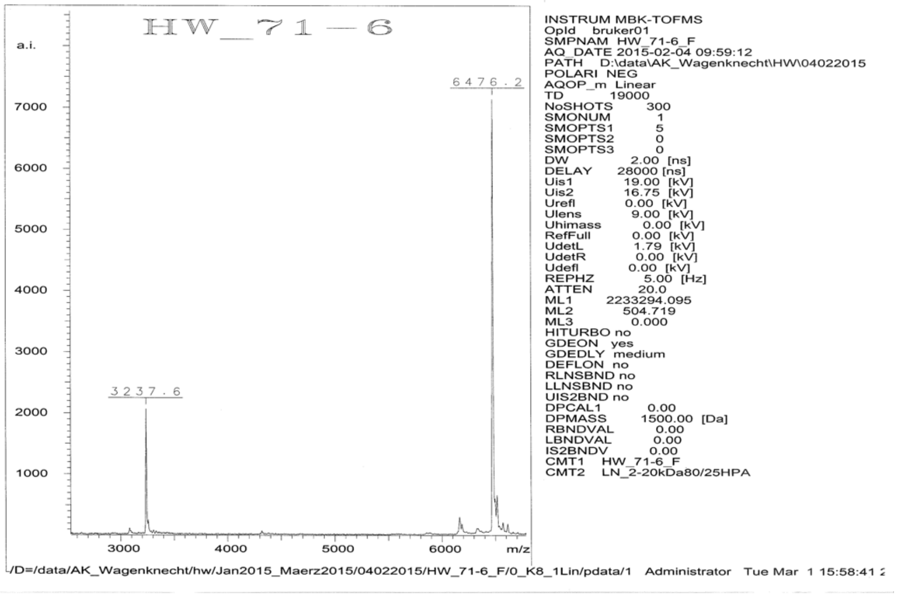

Figure S38: MALDI-TOF MS analysis of **DNA2aD3**; calculated: 6470.2 Da, found: 6476.2 Da.

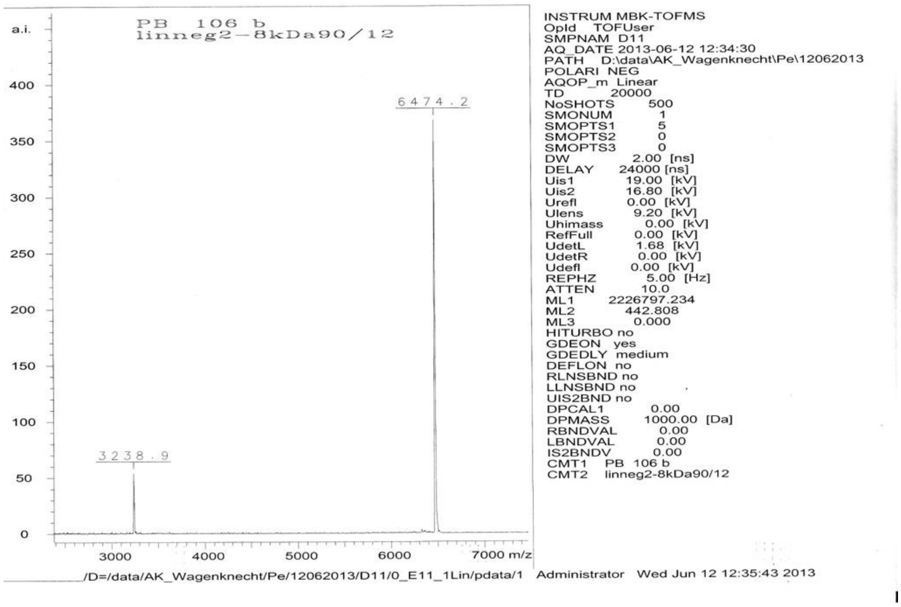

Figure S39: MALDI-TOF MS analysis of **DNA2rD3**; calculated: 6470.2 Da, found: 6474.2 Da.

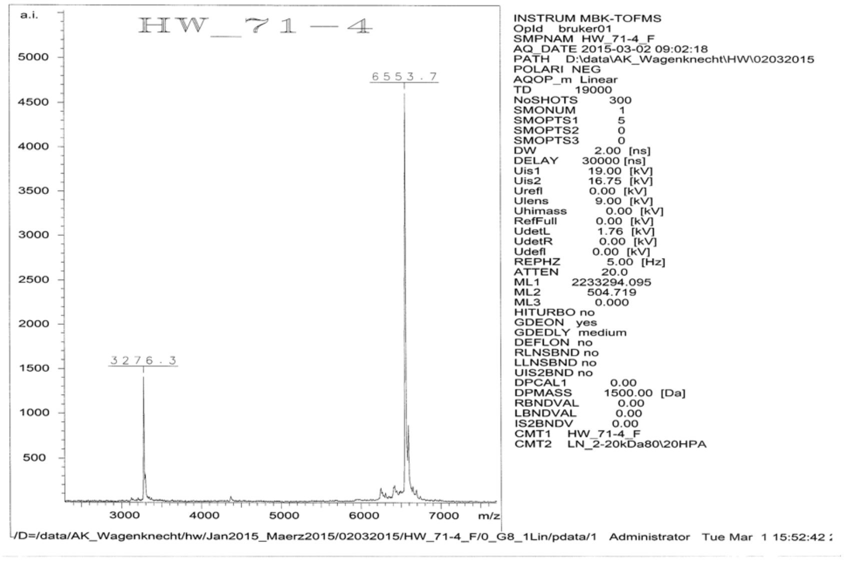

Figure S40: MALDI-TOF MS analysis of **DNA2aD4**; calculated: 6546.3 Da, found: 6553.7 Da.

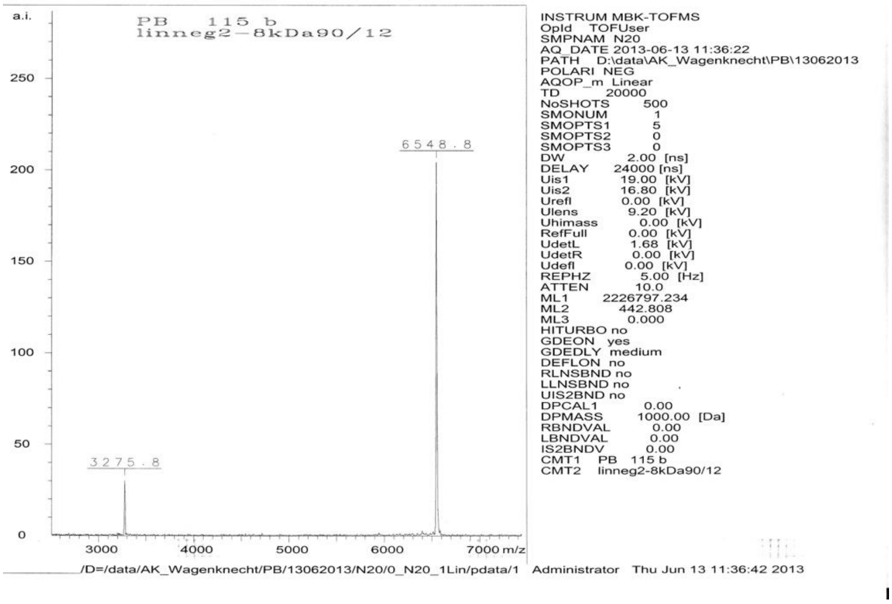

Figure S41: MALDI-TOF MS analysis of **DNA2rD4**; calculated: 6546.3 Da, found: 6548.8 Da.

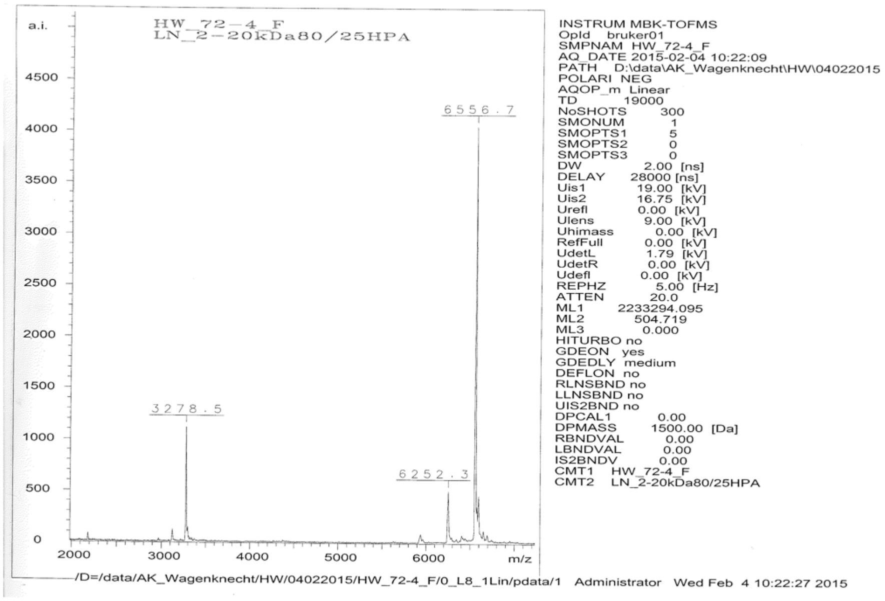

Figure S42: MALDI-TOF MS analysis of **DNA2aD5**; calculated: 6550.2 Da, found: 6556.7 Da.

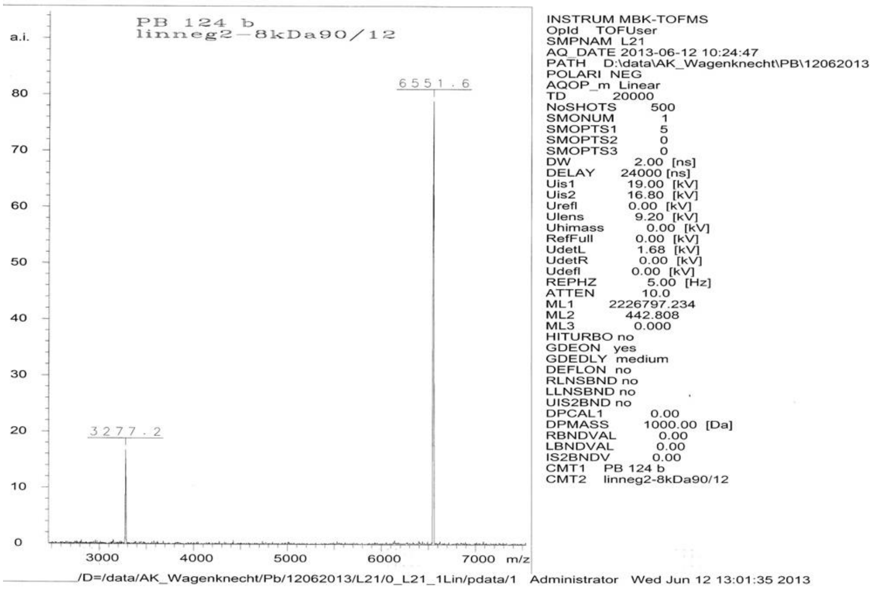

Figure S43: MALDI-TOF MS analysis of **DNA2rD5**; calculated: 6550.2 Da, found: 6551.6 Da.

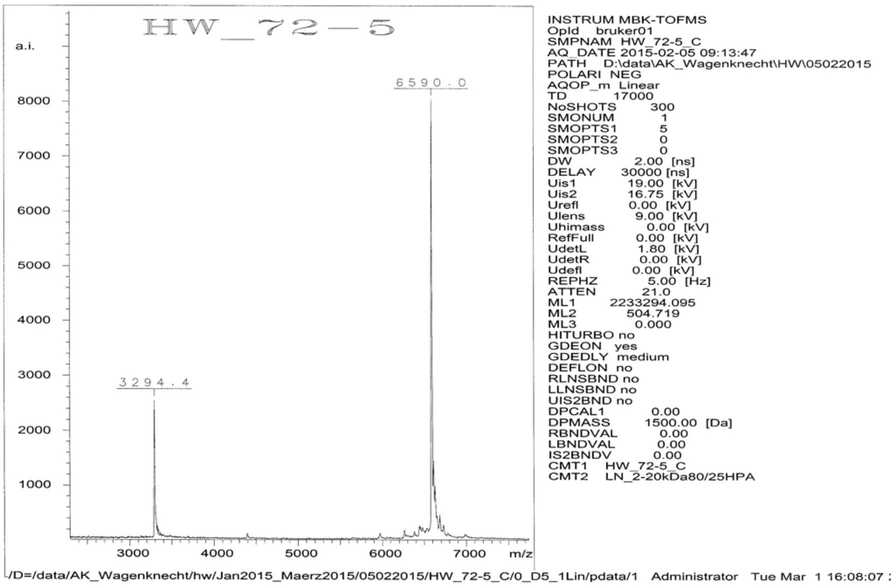

Figure S44: MALDI-TOF MS analysis of **DNA2aD6**; calculated: 6584.3 Da, found: 6590.0 Da.

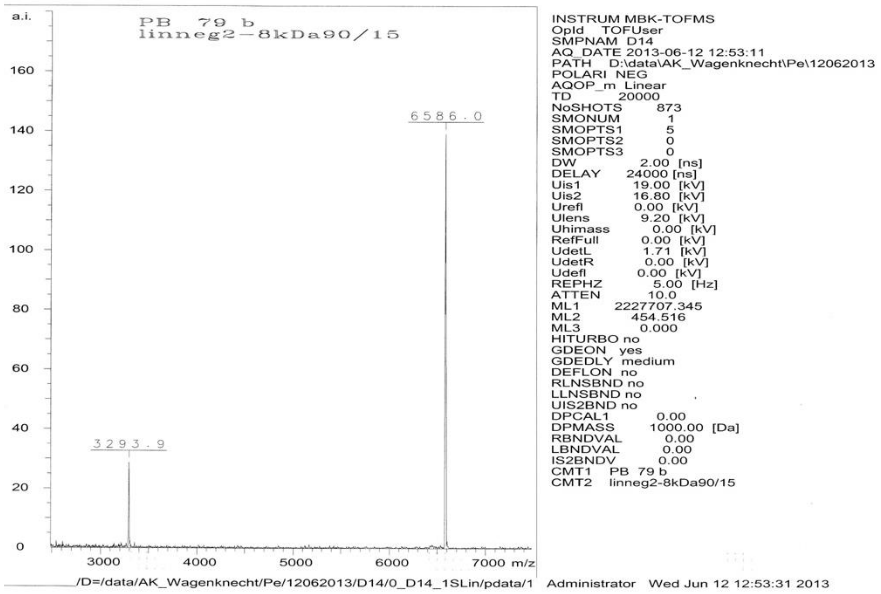

Figure S45: MALDI-TOF MS analysis of **DNA2rD6**; calculated: 6584.3 Da, found: 6586.0 Da.

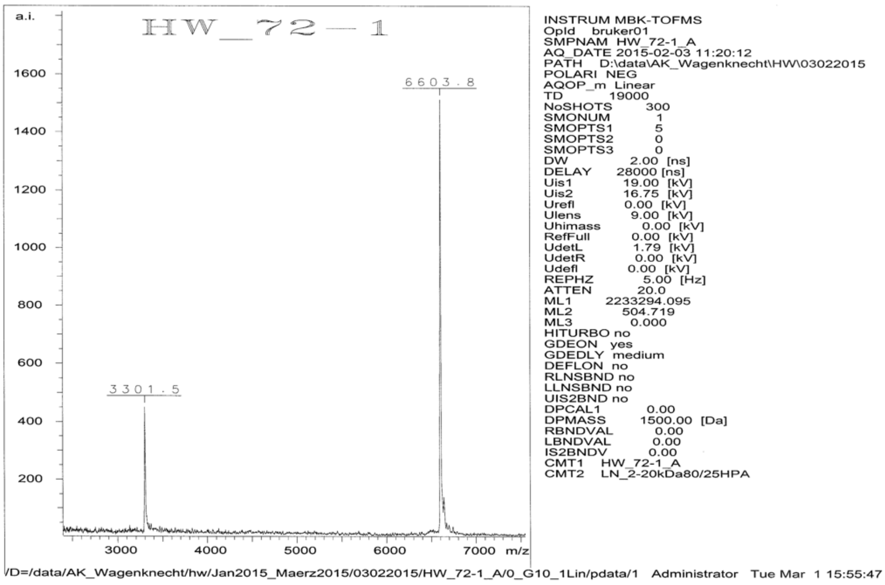

Figure S46: MALDI-TOF MS analysis of **DNA2aD7**; calculated: 6597.3 Da, found: 6603.8 Da.

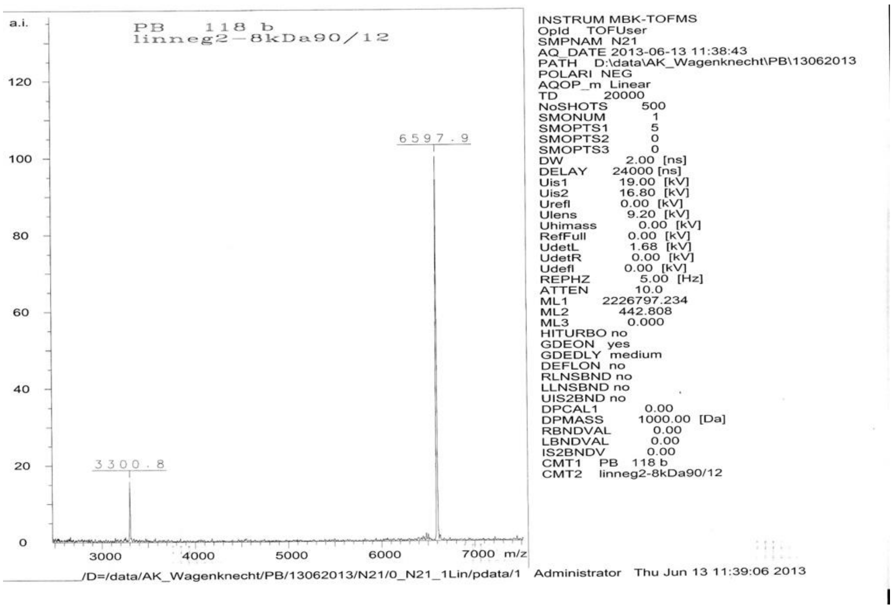

Figure S47: MALDI-TOF MS analysis of **DNA2rD7**; calculated: 6597.3 Da, found: 6597.9 Da.

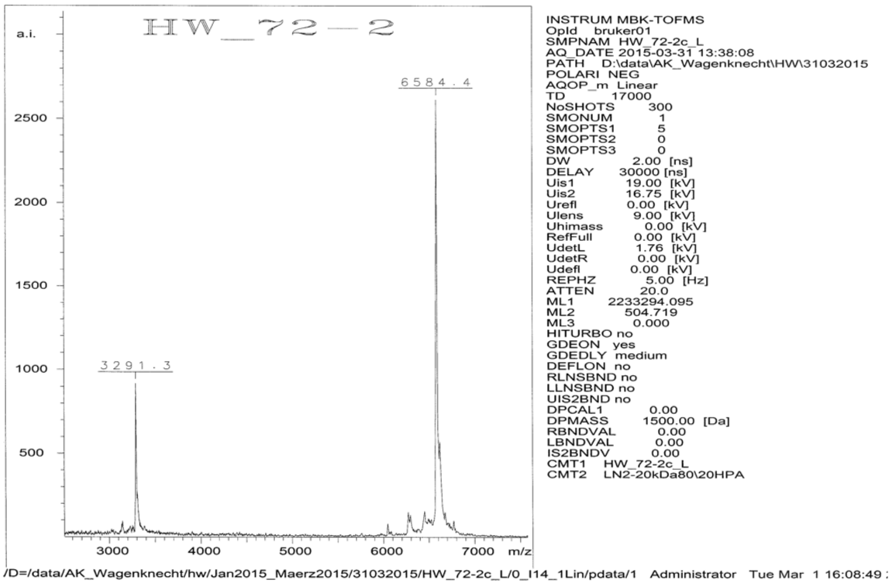

Figure S48: MALDI-TOF MS analysis of **DNA2aD8**; calculated: 6577.2 Da, found: 6584.4 Da.

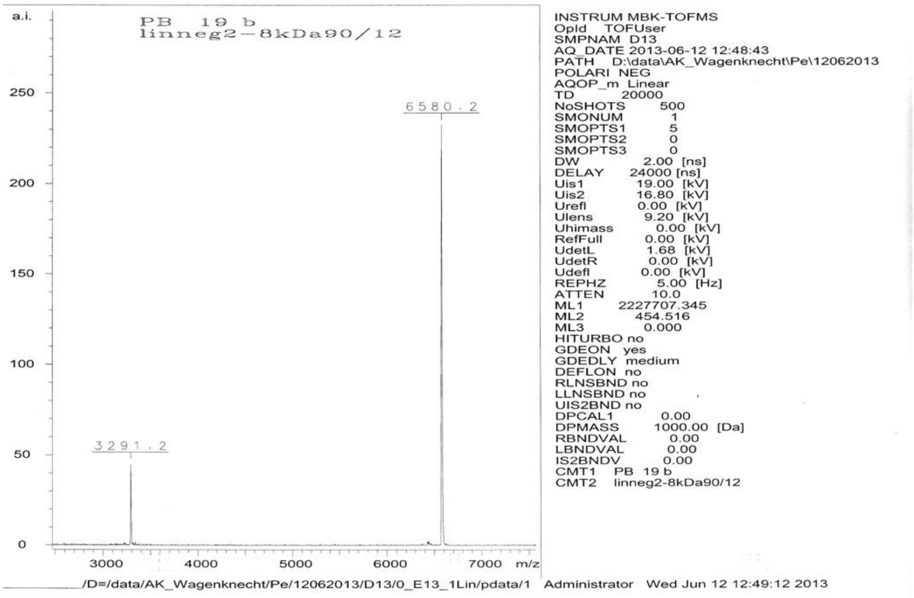

Figure S49: MALDI-TOF MS analysis of **DNA2rD8**; calculated: 6577.2 Da, found: 6580.2 Da.

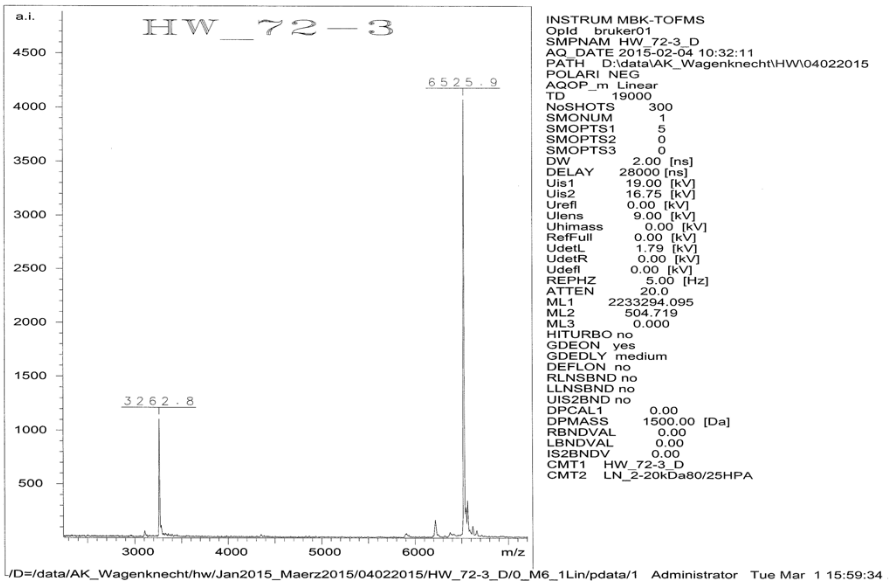

Figure S50: MALDI-TOF MS analysis of **DNA2aD9**; calculated: 6520.2 Da, found: 6525.9 Da.

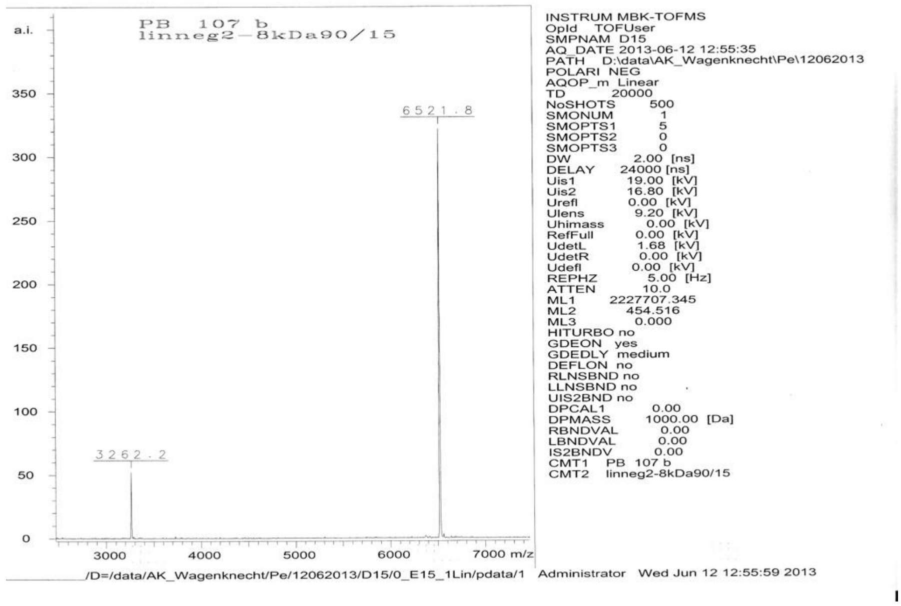

Figure S51: MALDI-TOF MS analysis of **DNA2rD9**; calculated: 6520.2 Da, found: 6521.8 Da.

4. Optical spectra for single-and double stranded modified DNA

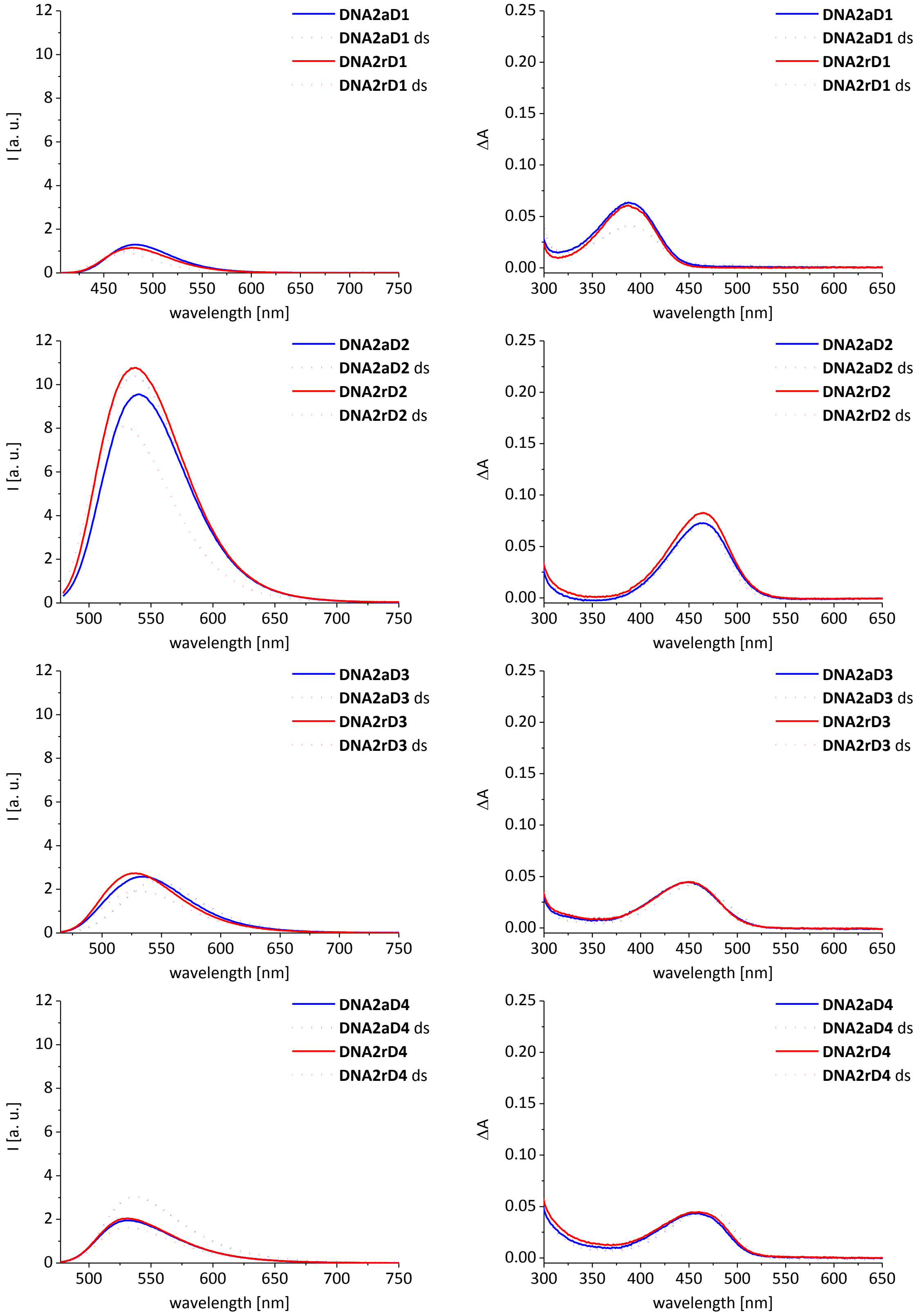

Figure S52: Fluorescence (left) and absorption (right) of single- (solid line) and double stranded (dotted line) DNA modified with **D1–D4**.

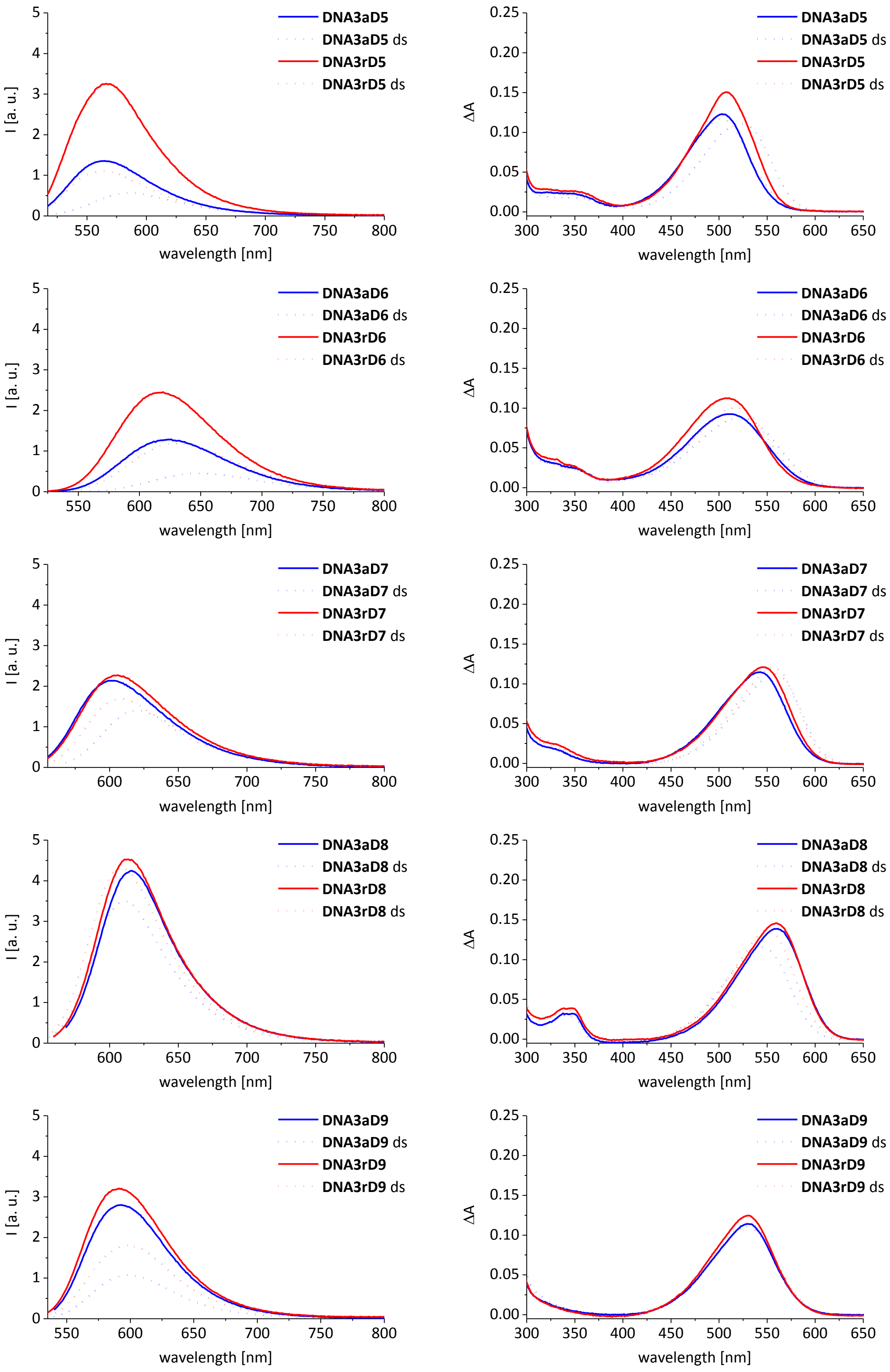

Figure S53: Fluorescence (left) and absorption (right) of single- (solid line) and double-stranded (dotted line) DNA modified with **D5–D9**.

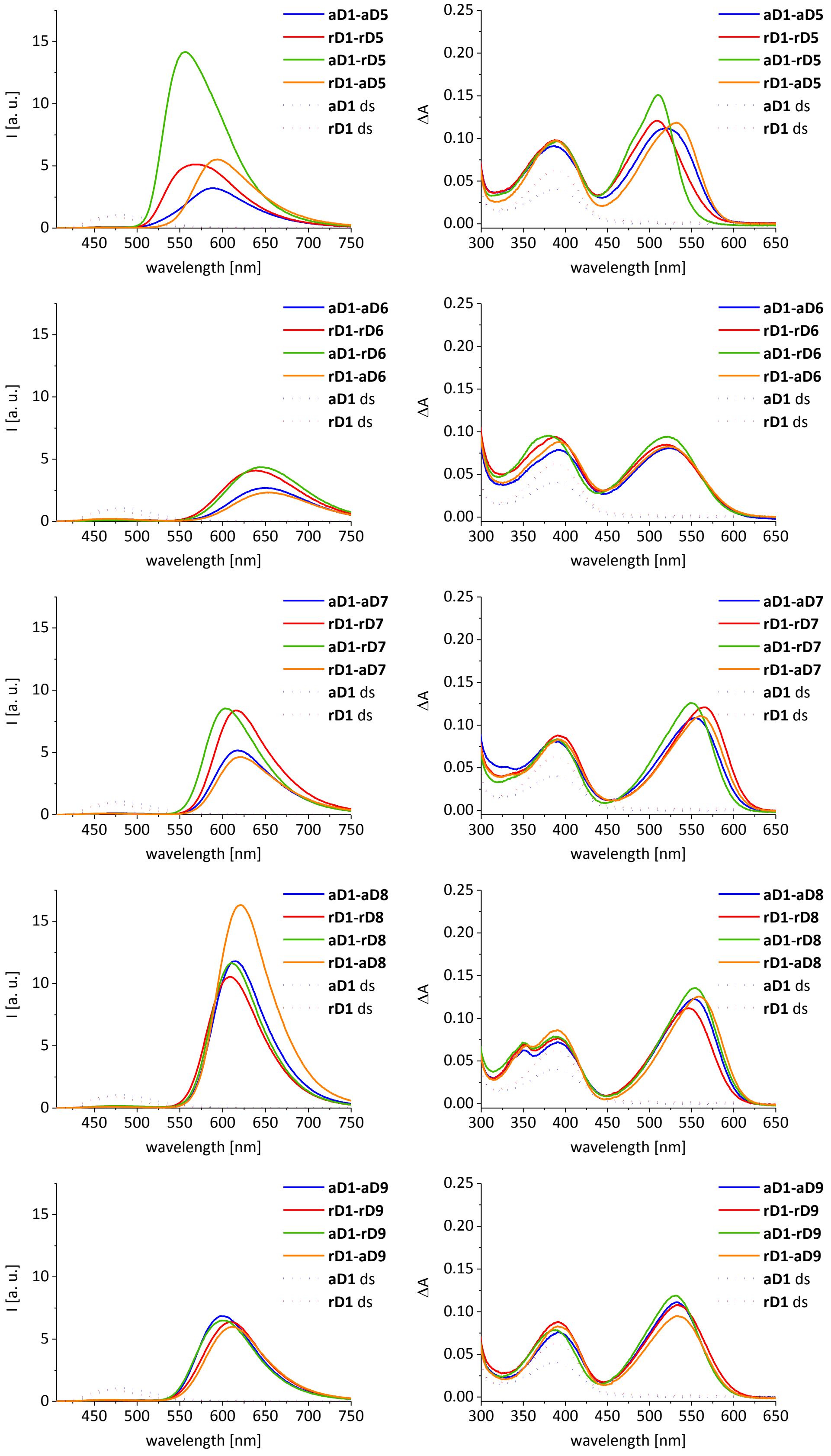

Figure S54: Fluorescence (left) and absorption (right) of single- (dotted line) and double-modified (solid line) DNA modified with **D1** as donor dye and **D5**–**D9** as acceptor dyes.

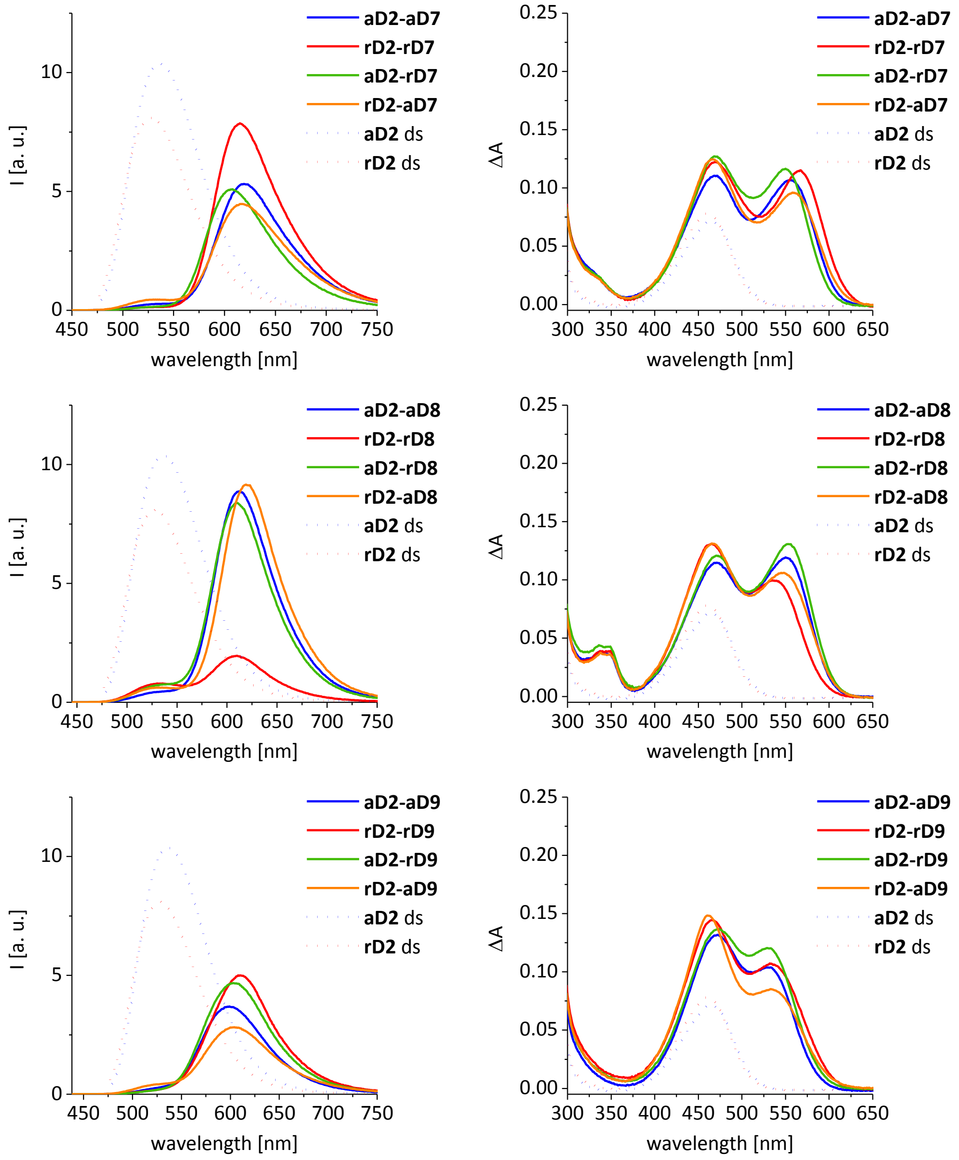

Figure S55: Fluorescence (left) and absorption (right) of single- (dotted line) and double-modified (solid line) DNA modified with **D2** as donor dye and **D7**–**D9** as acceptor dyes.

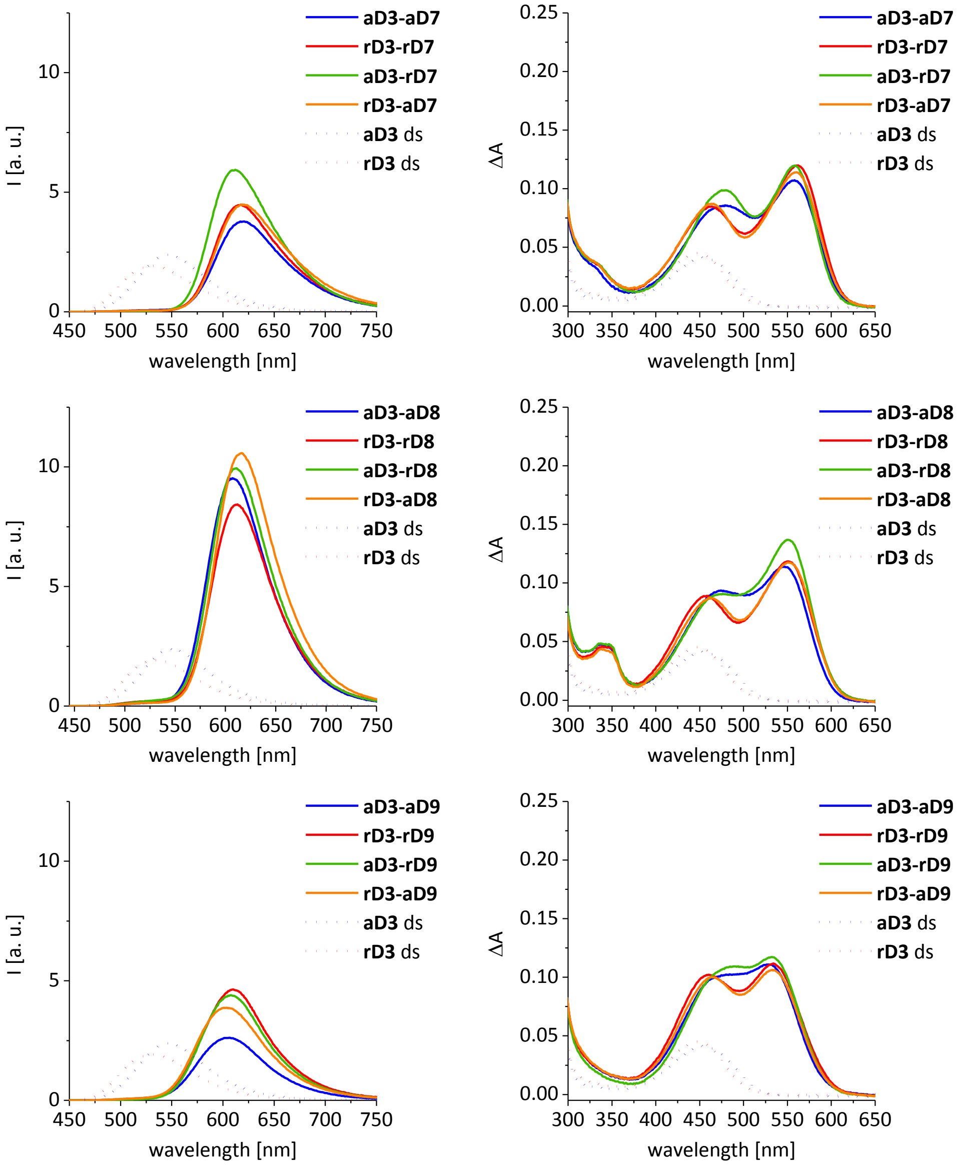

Figure S56: Fluorescence (left) and absorption (right) of single- (dotted line) and double-modified (solid line) DNA modified with **D3** as donor dye and **D7**–**D9** as acceptor dyes.

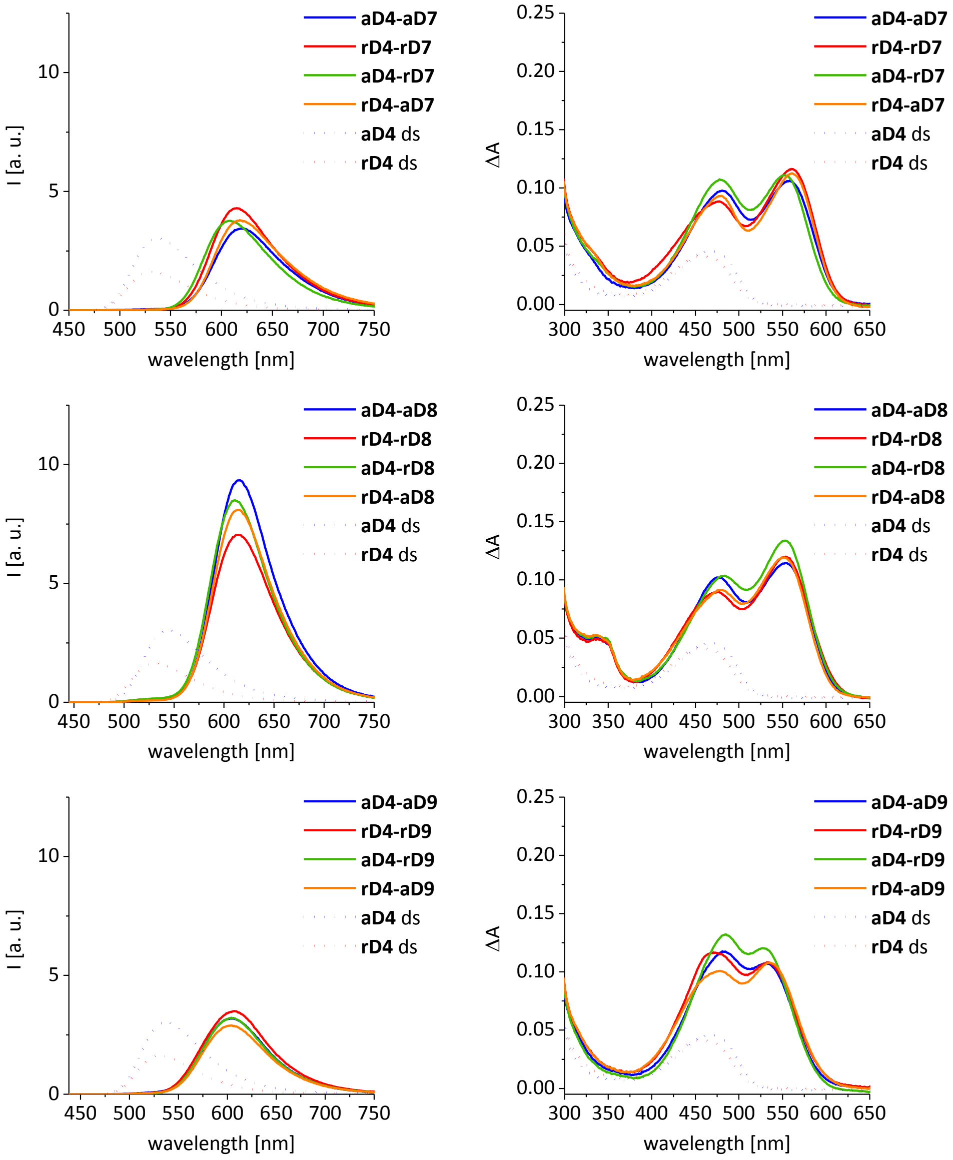

Figure S57: Fluorescence (left) and absorption (right) of single- (dotted line) and double-modified (solid line) DNA modified with **D4** as donor dye and **D7**–**D9** as acceptor dyes.

5. Melting temperatures of modified DNA double strands

Table S3: Melting temperatures T_m.

**DNA3a** and **DNA3r**		**DNA2a** and **DNA2r** **D1** T_m [°C]	**D2** T_m [°C]	**D3** T_m [°C]	**D4** T_m [°C]	**-** T_m [°C]
**D5**	**a-a**	63.0	-	-	-	67.1
	**a-r**	65.8	-	-	-	-
	**r-a**	67.6	-	-	-	-
	**r-r**	65.8	-	-	-	66.8
**D6**	**a-a**	61.5	-	-	-	63.9
	**a-r**	64.6	-	-	-	-
	**r-a**	64.5	-	-	-	-
	**r-r**	67.2	-	-	-	68.1
**D7**	**a-a**	62.1	63.8	67.1	66.5	66.5
	**a-r**	65.5	65.1	68.1	67.8	-
	**r-a**	65.4	66.2	67.7	68.2	-
	**r-r**	67.0	67.5	66.9	67.7	67.1
**D8**	**a-a**	63.5	64.5	68.0	67.2	66.5
	**a-r**	64.5	59.6	67.5	66.0	-
	**r-a**	68.2	67.7	69.0	69.0	-
	**r-r**	67.5	70.0	67.2	68.0	67.2
**D9**	**a-a**	61.3	62.5	64.4	68.2	63.5
	**a-r**	63.2	64.9	67.7	65.4	-
	**r-a**	64.2	65.3	65.2	66.1	-
	**r-r**	66.0	67.4	66.8	65.7	67.1

6. Additional cell images

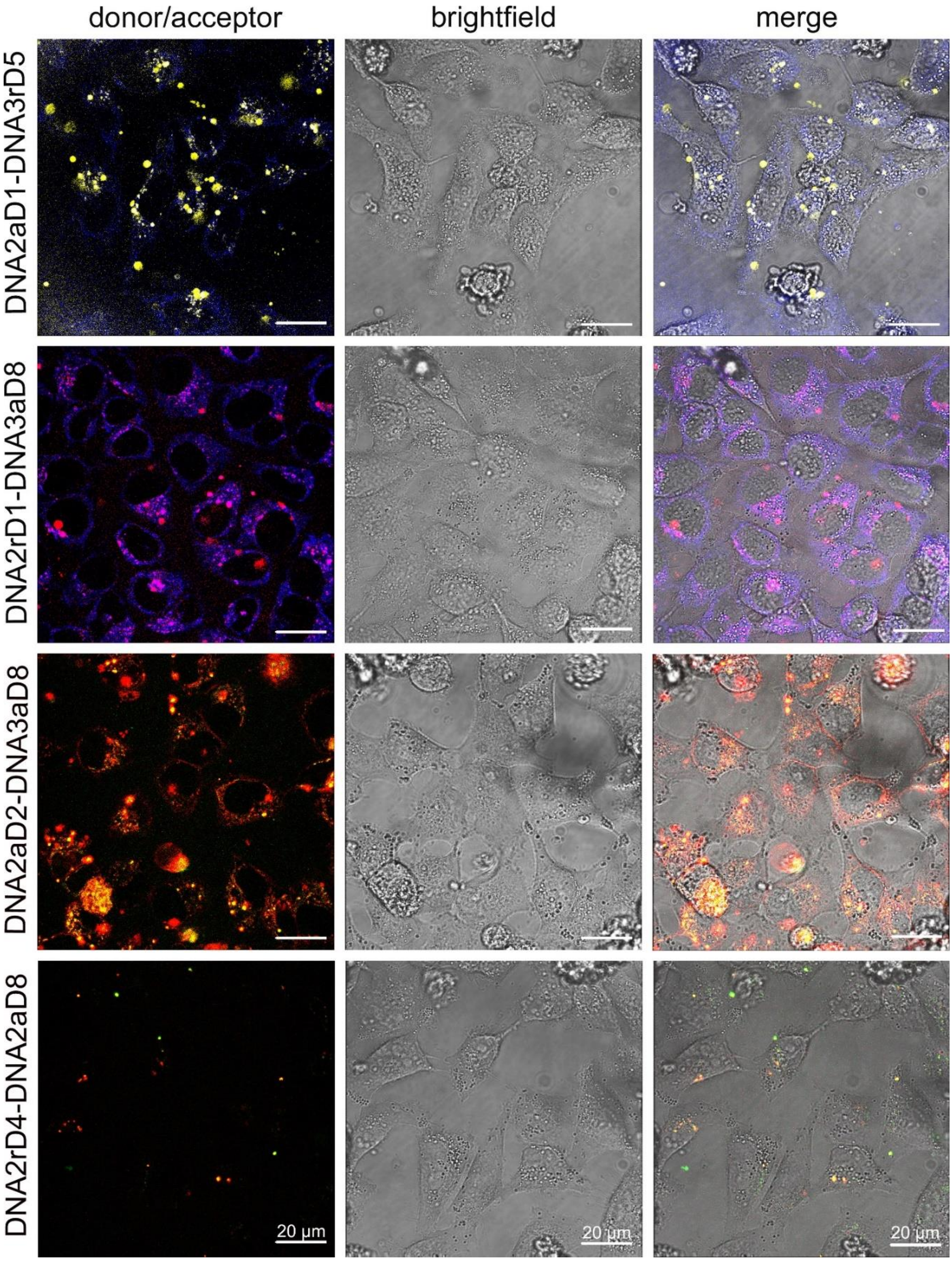

Figure S58: Confocal microscopy of HeLa cells after transfection with **DNA2aD1**–**DNA3rD5** (row 1), **DNA2rD1**–**DNA3aD8** (row 2), **DNA2aD2–DNA3aD8** (row 3) and **DNA2rD4**–**DNA2aD8** (row 4). The visualization was performed using a Leica TCS-SPE (DMi8) inverted microscope with an ACS APO 63×/1.30 oil objective. For **DNA2aD1**–**DNA3rD5** λ_{ex} = 405 nm (UV laser), λ_{em} = 435–470 nm (blue) and 575–750 nm (yellow), for **DNA2rD1**–**DNA3aD8** λ_{ex} = 405 nm (UV laser), λ_{em} = 415–550 nm (blue) and 575–750 nm (red), for **DNA2aD2–DNA3aD8** λ_{ex} = 488 nm (argon ion laser), λ_{em} = 490–550 nm (green) and 550–675 nm (red), for **DNA2rD4**–**DNA2aD8** λ_{ex} = 488 nm (argon ion laser), λ_{em} = 490–550 nm (green) and 675–800 nm (red).

6. Cytotoxicity in HeLa cells

To determine the toxicizy of the respective fluorophores (used in the DNA FRET pair constructs) in HeLa cells, the viability was tested using the CellTiter 96® Non-Radioactive Cell Proliferation Assay (Promega) according to the manufacturer's instructions. This assay is based on the intracellular reduction of a yellow tetrazolium salt (3-(4,5-dimethylthiazole-2-yl)-2,5-diphenyltetrazoliumbromide), MTT) into a violet formazan product by mitochondrial dehydrogenases, which only takes place in metabolic active cells. Therefore the amount of the generated formazan which can be determined by absorbance measurements is directly linked to cell viability.

1×10^4 HeLa cells were seeded in each well of a 96 well plate (Costar 3596, 96 Well Cell Culture Cluster, sterile) and cultured in 100 µL Dulbecco's modified Eagle's medium (DMEM, Gibco) supplemented with 10% fetal calf serum (FCS, Sigma-Aldrich) and 1% penicillin/streptomycin at 37 °C, 5% CO_2. After 24 hours the cells were treated with different concentrations of the dyes **D1**, **D2**, **D4**, **D5**, **D7** and **D8** (0.0375–0.30 µM). For each amount 6 wells were prepared and as a positive control (living cells) 6 wells were treated with respected DMSO concentrations to 0.30 µM dye samples. After an incubation time of 72 h another 6 wells were treated with 5 µL of 20% Triton X-100 (Serva) as a negative control for 100% dead cells for 5 min before all cells were treated with 15 µL of MTT reagent (Dye Solution according to manufacturer's instructions) per well and incubated for 2.5 h. Subsequently, 100 µL Solubilization Solution/Stop Mix was added to each well to lyze cells and dissolve the formed formazan crystals. After 24 h incubation at 37 °C the absorbance was measured at 595 nm using a 96-well plate reader (Ultra Microplate Reader ELx808, BioTEK Instruments, INC). Mean values and standard deviation were calculated from $n = 6$ experiments.

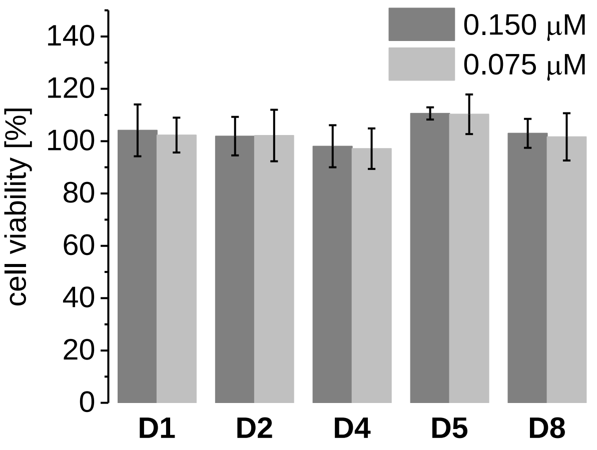

Figure S59: Cytotoxicity test with dyes **D1**, **D2**, **D4**, **D5** and **D8** that were applied for imaging experiments with live cells at two different concentrations (0.0150 µM and 0.075 µM).
